# High Add Valued Application of Turpentine in Crop Production through Structural Modification and QSAR Analysis

**DOI:** 10.3390/molecules23020356

**Published:** 2018-02-08

**Authors:** Yanqing Gao, Jingjing Li, Jian Li, Zhanqian Song, Shibin Shang, Xiaoping Rao

**Affiliations:** 1 Research & Development Center of Biorational Pesticide, College of Plant Protection, Northwest A&F University, Yangling 712100, Shaanxi, China; gaoyanqinggc@nwsuaf.edu.cn; 2 Shaanxi Province Key Laboratory of Economic Plant Resources Development and Utilization, College of Forestry, Northwest A&F University, Yangling 712100, Shaanxi, China; Lijingjing0355@163.com; 3 Institute of Chemical Industry of Forest Products, Chinese Academy of Forestry, Nanjing 210042, Jiangsu, China; lhssxly@hotmail.com (Z.S.); shangsb@hotmail.com (S.S.); rxping2001@163.com (X.R.)

**Keywords:** turpentine, herbicidal activity, SAR, QSAR

## Abstract

Turpentine is a volatile component of resin, which is an abundant forest resource in Southern China. As one of the most important components, the integrated application of β-pinene has been studied. The broad-spectrum evaluation of β-pinene and its analogues has, therefore, been necessary. In an attempt to expand the scope of agro-activity trials, the preparation and the evaluation of the herbicidal activity of a series of β-pinene analogues against three agricultural herbs were carried out. In accordance with the overall herbicidal activity, it is noteworthy that compounds **6k**, **6l**, and **6m** demonstrated extreme activity with IC_50_ values of 0.065, 0.065, and 0.052 mol active ingredients/hectare against *E. crus-galli*. The preliminary structure–activity relationship (SAR) was analyzed and the compounds with the appropriate volatility and substituent type that had beneficial herbicidal activity were analyzed. Simultaneously, the quantitative structure–activity relationship (QSAR) model was built and the most important structural features were indicated, which was, to a certain extent, in line with the SAR study. The study aimed to study the application of the forest resource turpentine in agriculture as a potential and alternative approach for comprehensive utilization.

## 1. Introduction

Weeds are considered worldwide as an infestation for crops. Some weed species interfere with the production of grain and vegetables and severely reduce the yield and quality [1]. Among them, *Echinochloa crus-galli* is one of the world’s worst weeds because it can seize the available soil nitrogen from the crop [2]. Effective and integrated control of these noxious weeds is essential for modern agriculture [3]. However, the wide use of single-type herbicides causes the serious occurrence of resistance weeds and a series of environmental issues [4]. Therefore, developing more effective and low toxic herbicides has become a priority for agriculture producers, researchers, and extension personnel [5,6,7,8,9,10]. As an important alternative to replacing traditional herbicides, forest resources have attracted increasing attention from researchers due to their unique qualities, such as being widely distributed, environmentally friendly, and diverse in chemical composition [11]. The acrylopimaric acid-based botanical herbicides have been synthesized from forest resource rosin [3]. The natural herbicide activity of *Satureja hortensis* L., an essential oil against two important weed species, has been reported [12]. Mansonone E has potential as a new natural pesticide for agricultural plant pathogen management [13]. Therefore, taking advantage of forest resources for agriculture through chemical structural modification is a viable solution for the production of new herbicides.

Resin, an abundant forest resource, is obtained from pines, which use it to resist external insect bites and other damage. In the growth of plants, resin components (including pinene) play many special roles, such as plant defense, plant interspecific phytochemical roles, attracting insects for pollination, and so on. Just as plant secondary metabolites, resin is agriculturally active and the high value-added application of resin is meaningful. In the complex chemical components of resin, turpentine, a monoterpene mixture of volatile oil, has excellent performance and potential for use in many fields. Turpentine has a high content of β-pinene and the cost to isolate β-pinene is low. However, studies on the biological activity of β-pinene derivatives are not rich. Therefore, further development of derivatives with bioactive properties using β-pinene as a starting material is gradually becoming a trend in turpentine processing and utilization. For instance, β-pinene and its analogues have agricultural activities, such as antifeedant, repellent, and antimicrobial properties [14,15,16]. The preparation of most compounds has been reported by us. Based on previous research, the reaction process has been improved. In order to further study the impact of heteroatoms on the activity, other novel heterocyclic compounds (**6k**–**6n**) have been synthesized [17,18]. The herbicidal activity of β-pinene and its analogues is worthy of study in order to develop some ecofriendly herbicides that can be applied in the control of noxious weeds.

In a program to design and synthesize herbicidal active substances via derivation of volatile β-pinene, there are many factors that need to be considered. First, the primary factor is the effective contact between the herbicide and the treated soil [19]. In order to improve the permeability (the penetration of the molecule through the plant epidermis, which is influenced by amphipathic characteristics, and better the biphasic solubility) and reduce volatilization, ester and amino groups were introduced into β-pinene. Second, new technology is needed to screen potential herbicides from mickle candidates for the weed control. QSAR, an efficient means to screen biological activity and mechanisms of action, was carried out through quantification software packages [20,21,22]. After the essential structural features for the activity have been defined, a study on the mechanisms of action should be targeted. The objective of this study was to continue expanding the application of volatile β-pinene in a high value-added field and to develop its broad-spectrum biological activity in agriculture.

## 2. Results and Discussion

### 2.1. Synthesis of Derivatives of β-Pinene

**Scheme 1:**
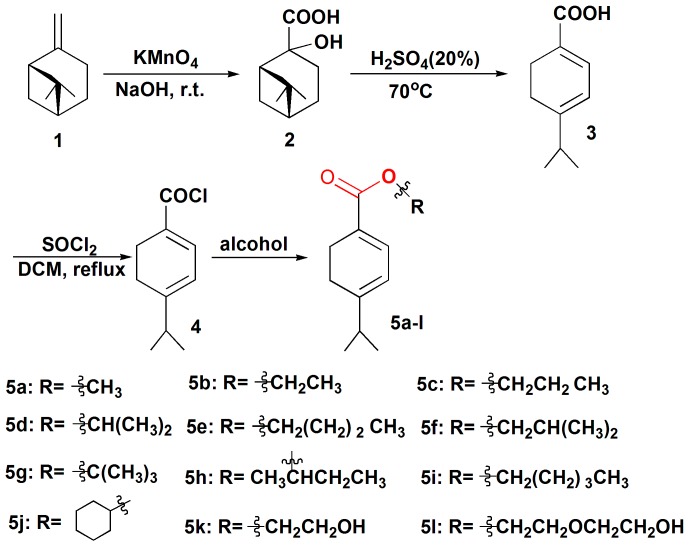
Structure and chemical synthesis of the compounds **5a**–**5l**.

**Scheme 2:**
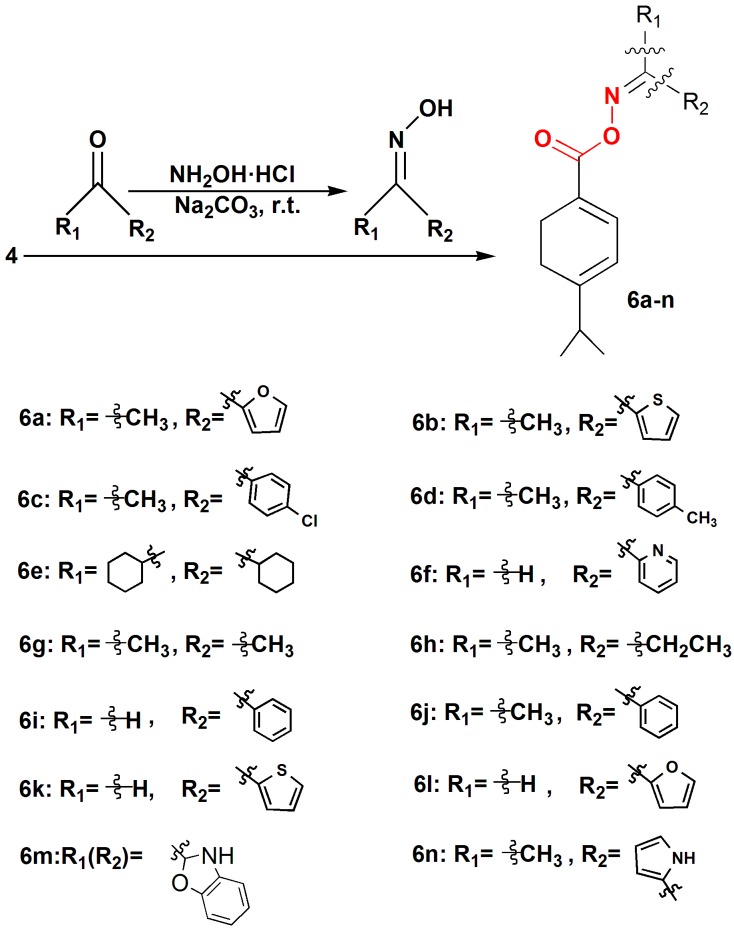
Structure and chemical synthesis of the compounds **6a**–**6n**.

The synthesis of two series of turpentine-based ester derivatives are shown in Scheme 1 and Scheme 2. The preparation of most of these compounds has been reported by us and, based on previous research, the reaction process has been improved. In order to further study the impact of heteroatoms on the activity, other novel heterocyclic compounds (**6k**–**6n**) were synthesized. The dehydrocumic acid was prepared from β-pinene through reactions of alkaline oxidation, dehydration, and isomerization, respectively. For the purpose of improving the reactivity of carboxylic acid, the dehydrocumic acid was activated by thionyl chloride into acid chloride. In the synthesis process of oxime esters, some of the intermediate oxime compounds have been prepared by us. In order to obtain a high yield, the synthesis process was optimized by regulating the reaction temperature and the reaction solvent and alkali. The optimal reaction conditions were ethanol as the reaction solvent, sodium carbonate as the base, and 25 °C as the reaction temperature. The structures of the title compounds have been well characterized by IR, ^1^H-NMR, MS, and elemental analysis.

### 2.2. Herbicidal Activity and Structure–Activity Relationships (SARs)

In Supplementary Materials (Table S1), the herbicidal activities of compounds **5a**–**5l** and **6a**–**6n** against *E. crus-galli*, *Amaranthus retroflexus*, and *Brassica campestris* L. *var. amplexicaulis Makino* were screened primarily at 200 g active ingredients/hectare. It is shown that all of the ester and oxime eater derivatives of β-pinene had good inhibition rates against *E. crus-galli* compared with another two herbs. From Table 1, the research on the herbicidal activity against *E. crus-galli* was done and the analogues displayed good herbicidal activity, which was more significant compared with compound **3**. It is noteworthy that compounds **6k**, **6l**, and **6m** demonstrated extreme activity with IC_50_ values of 0.065, 0.065, and 0.052 mol active ingredients/hectare against *E. crus-galli*, which were similar to that for sulfentrazone, a commercialized agriculture herbicide. For different types of β-pinene analogues, the level of herbicidal activity was oxime esters (**6a**–**6n**) > monoesters (**5a**–**5l**). Nevertheless, the environmental toxicology research on the newly synthesized compounds was very necessary. On the one hand, in order to avoid pesticide residues of herbicides in the soil, water, and air, the chemical stability and products of metabolism of the candidates must be experimentally tested under the influence of prolonged exposure to ambient temperature, sunlight, microflora or selected microorganisms, and plants. On the other hand, the impact of herbicides on non-target organisms such as bird, fish, and bees should also be assessed. Research in this area will be carried out in the follow-up work.

In the process of herbicides playing a role, there are many factors that restrict the interaction with the herbs. For instance, the suitable volatility and electrostatic interaction may be mainly affected [1,23]. In order to improve the volatility, the structure of the carboxylic acid (compound **3**) was modified with a hydroxyl group to obtain ester analogues. From the activity data shown in Table 1, the shorter branched chained compounds were intrinsically more toxic, for the monoesters compounds (**5a**–**5l**), and the activity sequence was **5a** > **5b** > **5c** > **5d** > **5g** > **5e** ≈ **5f** ≈ **5h** > **5i** > **5j** > **5k** > **5l**. In light of the point of view of Li et al. and Fan et al. aliphatic compounds are more volatile than aromatic compounds, and the volatility decreases with an increase in the molecular weight of the compounds [8,24]. Therefore, with regard to the same series of derivatives, along with the branched chain length increasing, the herbicidal activity showed a downward trend, and a similar trend was observed with oxime esters compounds (**6a**–**6n**). The electrostatic interaction of compounds is another important factor for herbicidal activity, and the type and position of the substituent has a great impact on the electrostatic effect of the compound. From investigations of the relationship of charge density and drug activity, the heteroatoms N, O, and S can play electron-withdrawing or electron-donating actions. Therefore, heterocyclic substituents such as thiophene, furan, and pyrrole were introduced to the derivatives. Based on the activity results, the rough order of herbicidal activity was aromatic substituted compounds was higher than aliphatic substituted compounds, and specifically heterocyclic substituted compounds (**6a**, **6b**, **6f**, **6k**, **6l**, **6m** and **6n**) was higher than benzene substituted compounds (**6c**, **6d**, **6i** and **6j**). As a preliminary conclusion, during the molecules playing an effect on *E. crus-galli*, the ring of thiophene and furan accepts the electron, which forms an electron transfer process with a protein or enzyme to achieve the herbicidal effect. To sum up, the volatility and substituent type are two important compound properties influencing the herbicidal activity.

### 2.3. QSAR Study on Herbicidal Activity Against E. crus-galli

**Figure 1:**
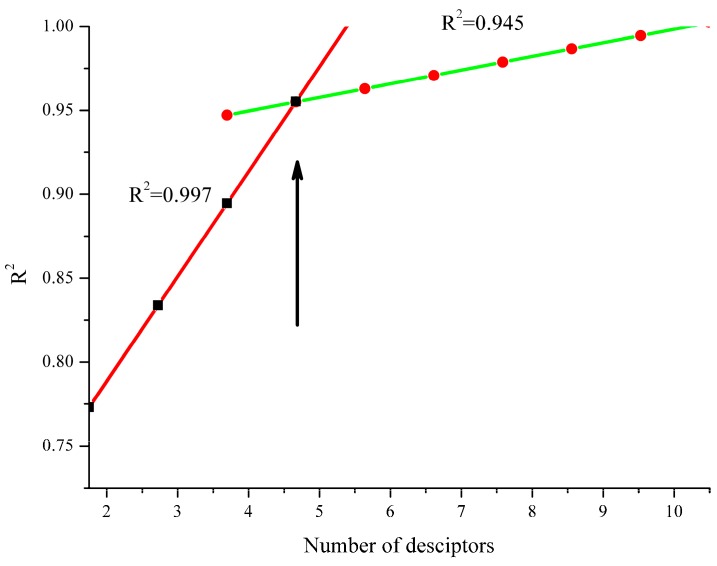
The “breaking point” rule results. The black code indicates a statistically notable improvement in the correlation coefficient, and the red code indicates it was not improving obviously.

There were six groups of descriptors that reflected all of the features of the compounds, such as molecular composition, connectivity, 3D-coordinates, charge distribution, and quantum chemical data. Through calculation and selection, several significant molecular descriptors were determined during the model development process. The most important descriptors in this work were the quantum-chemical descriptors. In an attempt to establish a relationship between activity and molecular descriptors through several regression approaches, the heuristic regression was chosen to obtain a QSAR model with satisfactory values of *R*^2^, *F*, and *S*^2^. Through the “breaking point” rule and the number of molecular descriptors being no more than one-third of the sample number plus one, the descriptor number was determined. The “breaking point” rule results are shown in Figure 1. Finally, the four-descriptor QSAR model was determined as the best model and is shown in Table 2. The four significant descriptors and their values are listed in the Supplementary Materials (Table S2).

**Figure 2:**
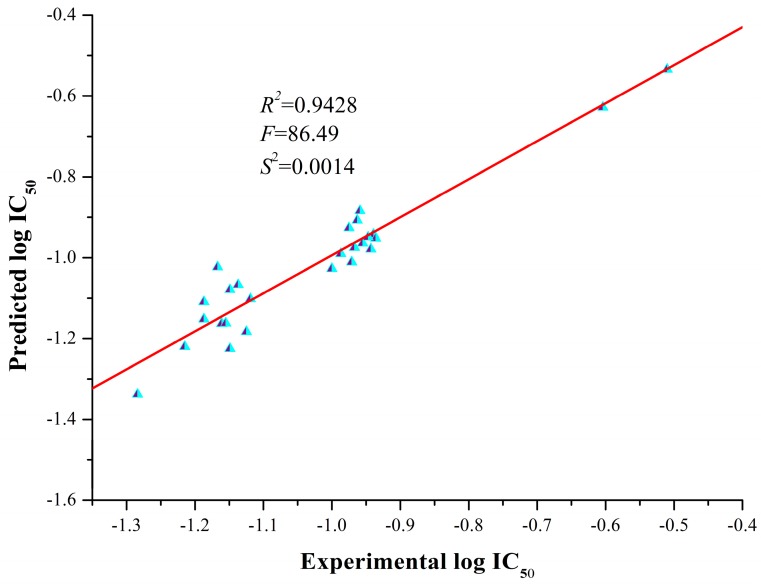
The comparison between experimental log IC_50_ and predicted log IC_50_.

The final four-descriptor QSAR model contained the sample number *N* = 26, and had the following statistical characteristics: *R*^2^ = 0.9428, *F* = 86.49, *S*^2^ = 0.0014. The experimental and predicted log IC_50_ values are shown in Table 3, which were also compared in Figure 2. The following equation (Equation (1)) describes the four-descriptor QSAR model. In the model, a positive sign (+) indicates that the descriptor value had a positive correlation with log IC_50_, and that the log IC_50_ value was higher as the descriptor value was larger. In contrast, a negative sign (−) indicates a negative correlation.

log IC_50_ = − 4.5445 − 0.0026 × Δ*H*_f_ + 2.8334 × *P_µµ_* + 0.0778 × *µ* − 0.3470 × *q*_max_° (1)

*N* = 26, *R*^2^ = 0.9428, *F* = 86.49, *S*^2^ = 0.0014

The validation of the established QSAR model was carried out by two validation methods. The internal validation method was described as the compounds **1**, **4**, **7**, etc. were assigned to group A; compounds **2**, **5**, **8**, etc. were assigned to group B. The other compounds belonged to subset C. The training set was two of these three groups, and the test set was the other group. The values of the corresponding test sets were predicted by the obtained correlation equation from the training set using the identical descriptors. The results of the internal validation are listed in Supplementary Materials (Table S3). The difference between *R*_Training_^2^ and *R*_Test_^2^ for the three sets was tiny enough to be ignored, and the average values of *R*_Training_^2^ and *R*_Test_^2^ were essentially identical to the overall *R*^2^ value, which meant the approving predictive power of the obtained model. In the “leave-one-out” method, the compounds **1**, **5**, **9**, etc. were the external test set and the others were the training set. The *R*^2^ value of the training set and test set were close and the QSAR developed in this study had good predictability.

With regard to the descriptors related to the QSAR model, the first important descriptor was the heat of formation of the molecule (Δ*H*_f_), which gives the energy of the molecule in the thermodynamic standard scale (elements in ideal gas state at 298.15 K and 101.325 Pa). It measures the change of enthalpy during the formation of 1 mol of the compound from its constituent elements with all substances in their standard states. This quantum-chemical descriptor indicates the level of reactivity of the molecule and can find the standard enthalpy change of any reaction [21]. The value of Δ*H*_f_ signifies the difficulty and heat change of the chemical reaction. A large absolute value Δ*H*_f_ implies a prone reaction and a negative value Δ*H*_f_ means an exothermic reaction. The converse is also true. From Equation (1), the Δ*H*_f_ value had a negative effect on log IC_50_ values.

**Figure 3:**
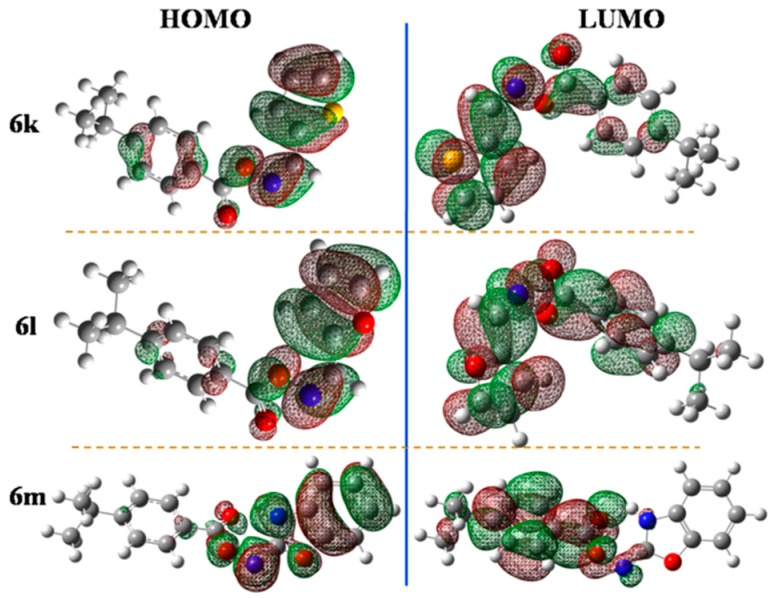
The HOMO and LUMO energy maps for compounds **6k**, **6l** and **6m**.

The second important descriptor is the max atomic orbital electronic population (*P_µµ_*), and it is a simplified index to describe the nucleophilicity of a molecule. According to the molecular orbital theory and frontier orbital theory, the highest occupied molecular orbital (HOMO) and the lowest unoccupied orbital (LUMO) are influential on the activity of the molecule [21]. When a molecule reacts with a target molecule, it occurs near the molecular front-orbital; therefore, the frontier orbital energy can greatly affect the herbicidal activity of a molecule. The suitable values of HOMO and LUMO are beneficial for the pesticide activity. From Figure 3, the frontier molecular orbitals (FMOs) of compounds **6k**, **6l**, and **6m** are shown, which indicate the highest occupied molecular orbital (HOMO) and the lowest unoccupied molecular orbital (LUMO). The HOMO and LUMO of the molecule mainly distribute in heterocyclic and benzene rings, which means that these active groups accept or provide electrons during the interaction with the receptor.

**Figure 4:**
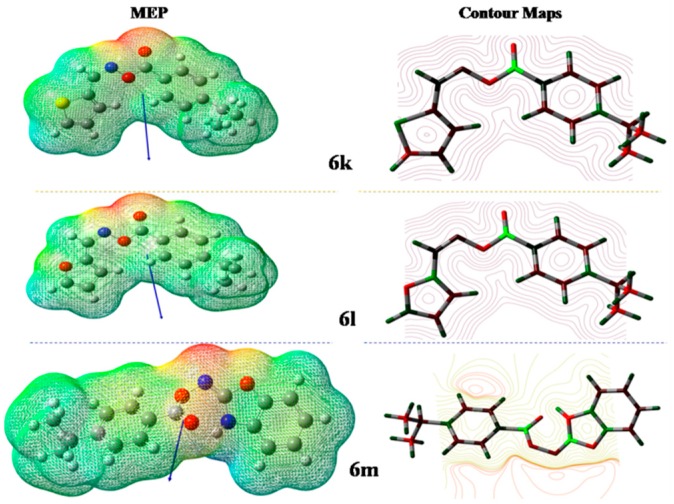
The molecular electrostatic potential and contour maps of compounds **6k**, **6l** and **6m**.

The third most important descriptor in the model is the tot dipole of the molecule, which measures the separation of positive and negative electrical charges within a molecule, that is, a measure of the overall polarity of the molecule [23,25,26]. The electric field strength of the dipole is proportional to the magnitude of the dipole moment. In Figure 4, the molecular electrostatic potential and contour maps of compounds **6k**, **6l** and **6m** are illustrated. The electron withdrawing substitute of O and S atoms, demonstrated by the green parts, represents the positive molecular orbitals. The electron donating substitute of N atoms shown in red, represents the negative molecular orbitals. From Equation (1), the μ value has a positive influence on the herbicidal activity.

**Figure 5:**
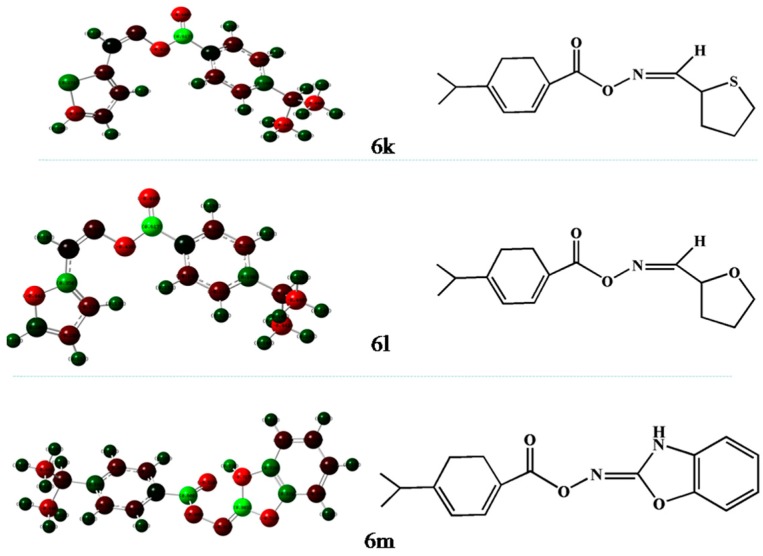
The optimized geometries and charge distribution of compounds **6k**, **6l** and **6m**.

The last important descriptor in the model is the max net atomic charge for an O atom. This electrostatic descriptor reflects the characteristics of the charge distribution of the molecule and is related to the strength of the intermolecular bonding interactions and characterizes the stability of the molecules, their conformational flexibility, and the other valency-related properties [24,25]. Figure 5 shows the optimized geometries and charge distribution of compounds **6k**, **6l** and **6m**. 

## 3. Materials and Methods 

### 3.1. Synthesis and Characterizations

β-pinene (**1**) was obtained from Jiangxi Jishui Hongda Natural Spices Ltd. (Jishui, China) of a commercial source. All the other chemicals used in the synthesis were of reagent grade and the purity was chemically pure (≥99.5%). A Nicolet IS 10 spectrophotometer (Thermo Nicolet Co., Ltd., Madison, WI, USA) was used for the Fourier transform infrared (FT-IR) spectra. A Bruker AV-300 nuclear magnetic resonance spectrometer (Bruker Co., Ltd., Karlsruhe, Germany) was used to measure the ^1^H-NMR spectra. An Agilent-5973 spectrophotometer was (Agilent Technologies Inc., Santa Clara, CA, USA) utilized for the MS spectra. A Bruker Q-TOF mass spectrometer (Bruker Bruker Co., Ltd., Karlsruhe, Germany) equipped with an electro spray ionization source was applied for ESI mass spectral data. Thin-layer chromatography (TLC) was employed to monitor the progress of reactions and determine the reaction’s end, which was carried out on Merck silica gel 60 GF254 plates (Qingdao Bangkai Hi-Tech Materials Co., Ltd., Qingdao, China) with eluent of petroleum ether/ethylacetate (*v*/*v* = 8:1). The preparation and the structures of title compounds are shown in Scheme 1 and Scheme 2.

#### 3.1.1. Synthesis of 4-Isopropylcyclohexa-1,3-dienecarboxylic acid (Dehydrocumic acid, **3**)

β-pinene was used as the raw material. Through alkaline oxidation, dehydration, and isomerism, the nopinic acid (**2**) and dehydrocumic acid (**3**) were prepared according to the method described previously [24]. During this process, the colorless crystal of **2** was obtained by recrystallization with organic solvent (such as ethanol or toluene) [25]. After dehydration and isomerization with concentrated sulfuric acid, the hydroxyl of the carboxylic acid was eliminated and the crude product **3** was obtained, which was used in the subsequent reaction after purification by recrystallization. Crystal Data for C_10_H_16_O_3_ (compound **2**, M = 184.23 g/mol): monoclinic, *a* = 26.796 (5) Å, *b* = 6.6560 (13) Å, *c* = 12.250 (3) Å, β = 112.23 (3) º, *V* = 2022.5 (9) Å^3^, *Z* = 8, *μ* = 0.009 mm^−1^, *T* = 293 K, *R* [*F*^2^ > 2σ (*F*^2^)] = 0.056, *wR* (*F*^2^) = 0.153, *S* = 1.00, *λ* = 0.71073 Å, *F*_000_ = 800.

#### 3.1.2. Synthesis of 4-Isopropylcyclohexa-1, 3-dienecarboxylates (Compounds **5a**–**5l**)

From the lead compound **3**, the 12 resulting compounds **5a**–**5l** were obtained by an esterification reaction between dehydrocumic acid chloride and the corresponding alcohols [26].

#### 3.1.3. Synthesis of Oximyl 4-Isopropylcyclohexa-1,3-dienecarboxylates (Compounds **6a**–**6n**)

From the lead compound **3**, the **14** resulting compounds **6a**–**6n** were obtained by an esterification reaction between dehydrocumic acid chloride and the corresponding oximes [8]. 

### 3.2. Herbicidal Activity Assay of β-Pinene Analogues

β-pinene analogues were evaluated for herbicidal activity of the representative monocotyledonous weed *E. crus-galli* and the dicotyledonous weeds *A. retroflexus* and *B. campestris* L. The herbs were grown in a greenhouse and the test method was the conventional plate method (in vitro) [25,26,27]. In the evaluation, all of the test compounds were dissolved in mixed solution of DMF and distilled water (*v*/*v* = 8:1). The inhibition percentage of seed germination was the mean value obtained through three independent experiments and the standard deviation (SD) values were limited within ±5%. On the basis of the series inhibition percentage, the IC_50_ results were calculated by the SPSS statistics program version 17.0 (SPSS Inc., Chicago, IL, USA). For stringency, the commercial herbicide sulfentrazone (Shanghai Aladdin Bio-Chem Technology Co., Ltd., Shanghai, China) was chosen as the positive control, and a pure solution without test compound was tested as the negative control.

The initial concentration was set at 200 g active ingredients/hectare and the lower required concentrations were diluted with water (100, 50, 25 and 12.5 g active ingredients/hectare). For better dispersion all the solutions were prepared with Tween (final concentration 0.1%) as surfactant. In petri dishes of 9 cm diameter, 2 pieces of filter paper were placed and 2 mL test compound solution with certain concentrations was sprayed evenly. For the accuracy of the results, the negative control was the same amount of DMF mixed with Tween-80 and water. Ten just-sprouting seeds of each weed species including *E. crus-galli*, *A. retroflexus* and *B. campestris* L. were grown in the test plate and allowed to germinate for 72 h at 28 ± 1 °C. Finally, the average values of the height of the ground portion of the above mentioned seedlings growing in each plate were calculated. For reproducibility and credibility, all of the tests at each concentration were repeated three times. The inhibition percentage (IP) was used to describe the control efficiency of the compounds and was calculated according to Equation (2):

IP (%) = [(*H*_0_ − *H*_1_)]/*H*_0_ × 100 (2)

where *H*_0_ is the height of control test and *H*_1_ is the height of treated test. Through regression analysis using SPSS Statistics 17.0, the IC_50_ values were calculated by the inhibition percentage of *E. crus-galli* at five concentrations for each test compound [28].

### 3.3. Building and Verification of the Quantitative Structure–Activity Relationship (QSAR) Model

The QSAR model was built and validated in accordance with the common procedure. Briefly, optimization of the most stable configuration was obtained with a Gaussian 03W package of programs (Gaussian Inc., Wallingford, CT, USA,), and all the molecular descriptors were calculated by CODESSA 2.7.15 (The copyright of this version is held by the Center of Heterocyclic Chemistry, University of Florida.) [29]. The “breaking point” rule for determining the number of the descriptors is described in Figure 1. The best multilinear regression showed a significant increase in *R*^2^ when the number of the descriptors was less than or equal to 4. However, there was negligible change in *R*^2^ when the number of descriptors increased from 4 to 5. Descriptors with high t values were accepted and those with low t values were rejected. A “breaking point” indicates that the improvement of the regression model has become non-significant (*R*^2^ < 0.02-0.04). For the determination of the most significant structural features of herbicidal activity against *E. crus-galli*, the heuristic analysis was to build the QSAR model. During the model development process, the related statistical parameters (*R*^2^, *S*^2^ and *F*) were determined to assess the predictability of a given model. In this study, a model displaying good predictability between the compounds’ structures and the log IC_50_ values was developed. To ensure high quality of the final QSAR results, two verification methods, internal validation, and the “leave-one-out” cross-validation were used for determination [30].

## 4. Conclusions

In order to expand the efficient application of the forest resource β-pinene to agriculture, two series of β-pinene analogues—ester and oxime esters—were prepared with suitable volatility and a substituent type through structural modification. The herbicidal activity against *E. crus-galli*, *A. retroflexus* and *B. campestris* L. was evaluated. Among them, the activity against *E. crus-galli* was more ideal; therefore, a study on the structure–activity relationship (SAR) against *E. crus-galli* was focused on. The preliminary conclusion is that compounds with esterification modification and a short chain length are beneficial for herbicidal activity. In addition, the heteroatoms N, O and S can play an active role. Simultaneously, the quantitative structure–activity relationship (QSAR) model (*R*^2^ = 0.9428, *F* = 86.49, *S*^2^ = 0.0014) was built and indicates that the most important structural feature is the heat of formation of the molecule (Δ*H*_f_). In view of these results, for the further exploitation of ecofriendly herbicides and the better application of volatile β-pinene, short chain esters, and heterocyclic-substituted derivatives could be the direction of the future development of new structures. It is noteworthy that environmental toxicology should be focused on in future work.

## Figures and Tables

**Table 1:** Herbicidal activity of compounds against *E. crus-galli* in a seed germination method.

Compd.	Herbicidal Activity at a Concentration of (g Active Ingredients/Hectare)					IC_50_		*y* = *a* + *bx*	*R*^2^	log IC_50_
	200	100	50	25	12.5	g Active Ingredients/Hectare	mol Active Ingredients/Hectare			
**3**	80	68	55	45	32	49.880	0.300	*y* = −0.643 + 0.011*x*	0.962	−0.523
**5a**	100	91	72	57	42	19.886	0.110	*y* = −0.617 + 0.031*x*	0.991	−0.959
**5b**	100	90	71	56	41	21.204	0.109	*y* = −0.643 + 0.030*x*	0.989	−0.963
**5c**	100	90	70	55	41	22.024	0.106	*y* = −0.667 + 0.030*x*	0.993	−0.975
**5d**	100	90	70	54	40	23.099	0.111	*y* = −0.712 + 0.031*x*	0.993	−0.955
**5e**	96	80	68	50	39	25.505	0.115	*y* = −0.498 + 0.020*x*	0.983	−0.939
**5f**	90	78	64	50	39	25.679	0.116	*y* = −0.373 + 0.015*x*	0.949	−0.936
**5g**	100	90	69	53	40	23.908	0.108	*y* = −0.736 + 0.031*x*	0.996	−0.967
**5h**	91	78	65	49	40	25.335	0.114	*y* = −0.383 + 0.015*x*	0.959	−0.943
**5i**	92	81	64	50	38	26.605	0.113	*y* = −0.442 + 0.017*x*	0.951	−0.947
**5j**	88	76	60	50	40	27.573	0.103	*y* = −0.360 + 0.013*x*	0.959	−0.987
**5k**	75	60	49	42	35	64.910	0.309	*y* = −0.570 + 0.009*x*	0.972	−0.510
**5l**	80	60	48	41	35	63.125	0.249	*y* = −0.656 + 0.010*x*	0.994	−0.604
**6a**	100	96	77	61	40	18.905	0.069	*y* = −0.768 + 0.041*x*	0.990	−1.161
**6b**	100	96	76	61	43	17.510	0.061	*y* = −0.674 + 0.038*x*	0.994	−1.215
**6c**	95	84	65	58	37	21.708	0.068	*y* = −0.416 + 0.019*x*	0.954	−1.167
**6d**	93	81	64	55	40	21.115	0.071	*y* = −0.345 + 0.016*x*	0.965	−1.149
**6e**	85	74	59	50	41	26.975	0.076	*y* = −0.305 + 0.011*x*	0.950	−1.119
**6f**	100	95	74	60	40	19.723	0.073	*y* = −0.738 + 0.037*x*	0.989	−1.137
**6g**	90	76	61	54	40	23.711	0.107	*y* = −0.323 + 0.014*x*	0.967	−0.971
**6h**	88	73	60	54	41	23.411	0.100	*y* = −0.282 + 0.012*x*	0.969	−1.000
**6i**	98	85	69	57	40	20.229	0.075	*y* = −0.460 + 0.023*x*	0.990	−1.125
**6j**	93	79	63	55	42	20.196	0.071	*y* = −0.314 + 0.016*x*	0.982	−1.149
**6k**	100	98	78	60	45	17.182	0.065	*y* = −0.741 + 0.043*x*	0.993	−1.187
**6l**	100	97	79	63	44	16.004	0.065	*y* = −0.665 + 0.042*x*	0.995	−1.187
**6m**	100	95	77	60	46	15.714	0.052	*y* = −0.564 + 0.036*x*	0.998	−1.284
**6n**	100	95	75	61	40	19.077	0.070	*y* = −0.719 + 0.038*x*	0.987	−1.155
Sulfentrazone	100	80	69	55	39	15.743	0.041	*y* = −0.820 + 0.052*x*	0.999	−1.387

Values in columns followed by similar letters were not significantly different according to Fisher’s protected; LSD test (*P* = 0.05).

**Table 2:** The best four-descriptor model.

Descriptor No.	*X*	±Δ*X*	*t*-Test	Descriptor
0	−4.5445	7.3386 × 10^−1^	−6.1926	Intercept
1	−2.5592 × 10^−3^	2.3118 × 10^−4^	−11.0701	Δ*H*_f_^a^
2	2.8334	3.8752 × 10^−1^	7.3116	*P_µµ_* ^b^
3	7.7801 × 10^−2^	1.0981 × 10^−2^	7.0850	*µ* ^c^
4	−3.4698 × 10^−1^	1.5347 × 10^−1^	−2.2609	*q*_max_° ^d^

^a^ Final heat of formation of atoms. ^b^ Max atomic orbital electronic population. ^c^ Tot dipole of the molecule. ^d^ Max net atomic charge for a O atom.

**Table 3:** The difference between the experimental log IC_50_ and predicted log IC_50_.

No.	Compd.	Calc. Log IC_50_	Exp. Log IC_50_	Difference
1	**5a**	−0.884	−0.959	0.075
2	**5b**	−0.907	−0.963	0.056
3	**5c**	−0.926	−0.975	0.049
4	**5d**	−0.964	−0.955	−0.009
5	**5e**	−0.942	−0.939	−0.003
6	**5f**	−0.952	−0.936	−0.016
7	**5g**	−0.975	−0.967	−0.008
8	**5h**	−0.979	−0.943	−0.036
9	**5i**	−0.949	−0.947	−0.002
10	**5j**	−0.990	−0.987	0.003
11	**5k**	−0.534	−0.510	−0.024
12	**5l**	−0.628	−0.604	−0.024
13	**6a**	−1.163	−1.161	−0.002
14	**6b**	−1.220	−1.215	−0.005
15	**6c**	−1.023	−1.167	0.044
16	**6d**	−1.079	−1.149	0.070
17	**6e**	−1.102	−1.119	0.017
18	**6f**	−1.067	−1.137	0.070
19	**6g**	−1.011	−0.971	−0.040
20	**6h**	−1.027	−1.000	−0.027
21	**6i**	−1.183	−1.125	−0.058
22	**6j**	−1.225	−1.149	−0.076
23	**6k**	−1.152	−1.187	0.035
24	**6l**	−1.109	−1.187	0.078
25	**6m**	−1.338	−1.284	−0.054
26	**6n**	−1.162	−1.155	−0.007

## Supplementary Materials

The following are available online. IR, ^1^H-NMR, MS and elemental analysis data for the target compounds. The herbicidal activity and structure descriptors of the title compounds are also available as supplementary information.
