# Supplementary material for: High Add Valued Application of Turpentine in Crop Production through Structural Modification and QSAR Analysis
Source: Molecules. 2018 Feb 8;23(2):356. doi: 10.3390/molecules23020356 (PMC6017721; doi:10.3390/molecules23020356)
Supplement: Supplementary file 1 [file molecules-23-00356-s001.zip › Supplementary Materials.docx]

High Add Valued Application of Turpentine in Crop Production through Structural Modification and QSAR Analysis

Yanqing Gao ^1,†^, Jingjing Li ^2,†^, Jian Li ^2,*^, Zhanqian Song ^3^, Shibin Shang ^3^ and Xiaoping Rao ^3^

^1^ College of Plant Protection, Northwest A&F University, Yangling, Shaanxi 712100, China; gaoyanqinggc@nwsuaf.edu.cn (Y.G.)

^2^ College of Forestry, Northwest A&F University, Yangling, Shaanxi 712100, China; Lijingjing0355@163.com (J.L.); ericlee99@nwsuaf.edu.cn (J.L.)

^3^ Institute of Chemical Industry of Forest Products, Chinese Academy of Forestry, Nanjing, Jiangsu 210042, China; lhssxly@hotmail.com (Z.S.); shangsb@hotmail.com (S.S.); rxping2001@163.com (X.R.)

***** Corresponding author: [ericlee99@nwsuaf.edu.cn](mailto:ericlee99@nwsuaf.edu.cn)

^†^ These authors contributed equally to this paper and share co-first authorship.

**1. Physico-Chemical Characteristics of the Synthesized Compounds**

**4-Isopropylcyclohexa-1, 3-dienecarboxylic acid (3, C_10_H_14_O_2_)**

Yield: 4.27 g (70.0%); white solid; m.p. 130 °C. IR (cm^-1^): 3415 (-OH); 2961, 2874 (-CH_3_, -CH_2_); 1700 (C=O). ^1^H- NMR (CDCl_3_, *δ*/ppm, 300 MHz): 6.94 (d, 1H, O=C-C=CH-); 5.90 (d, 1H, -CH=C-CH-(Me)_2_); 2.46 (m, 1H, -CH-(Me)_2_); 2.51 (t, 2H, O=C-C-CH_2_-); 2.27 (t, 2H, O=C-C-CH_2_-CH_2_-); 1.10 (d, 6H, -CH_3_). TOF-ESI-MS (m/z): 167 [M + H] ^+^. Anal. calcd for C_10_H_14_O_2_: C, 72.26; H, 8.49. Found: C, 72.21; H, 8.59.

**Methyl 4-isopropylcyclohexa-1, 3-dienecarboxylate (5a, C_11_H_16_O_2_)**

Yield: 3.24 g (60.0%); yellow liquid. IR (cm^-1^): 2960, 2873 (-CH_3_, -CH_2_); 1709 (C=O); 1273, 1088 (C-O-C). ^1^H-NMR (CDCl_3_. δ/ppm. 300 MHz): 6.99, 7.00 (d, 1H, O=C-C=CH-); 5.81-5.83 (d, 1H, -CH=C-CH-(Me)_2_); 3.92 (s, 3H, -OCH_3_); 2.91-3.00 (m, 1H, -CH-(Me)_2_); 2.41-2.48 (t, 2H, O=C-C-CH_2_-); 2.16-2.23 (t, 2H, O=C-C-CH_2_-CH_2_-); 1.25, 1.28 (d, 6H, -CH_3_). TOF-ESI-MS (m/z): 179 [M - H] ^-^; 181 [M + H] ^+^. Anal. calcd for C_11_H_16_O_2_: C, 73.30; H, 8.95. Found: C, 73.11; H, 9.00.

**Ethyl 4-isopropylcyclohexa-1, 3-dienecarboxylate (5b, C_12_H_18_O_2_)**

Yield: 3.81 g (65.5%); yellow liquid. IR (cm^-1^): 2961, 2872 (-CH_3_, -CH_2_); 1708 (C=O); 1263, 1093 (C-O-C). ^1^H- NMR (CDCl_3_. δ/ppm. 300 MHz): 6.99, 7.00 (d, 1H, O=C-C=CH-); 5.80-5.82 (d, 1H, -CH=C-CH-(Me)_2_); 4.17-4.24 (q, 2H, -OCH_2_-); 2.91-3.00 (m, 1H, -CH-(Me)_2_); 2.41-2.48 (t, 2H, O=C-C-CH_2_-); 2.16-2.22 (t, 2H, O=C-C-CH_2_-CH_2_-); 1.34-1.44 (t, 3H, O-CH_2_-CH_3_); 1.25, 1.28 (d, 6H, -CH_3_). TOF-ESI-MS (m/z): 193 [M - H] ^-^; 195 [M + H] ^+^. Anal. calcd for C_12_H_18_O_2_: C, 74.19; H, 9.34. Found: C, 74.11; H, 9.38.

**Propyl 4-isopropylcyclohexa-1, 3-dienecarboxylate (5c, C_13_H_20_O_2_)**

Yield: 5.00 g (80.2%); yellow liquid. IR (cm^-1^): 2962, 2876 (-CH_3_, -CH_2_); 1704 (C=O); 1260, 1083 (C-O-C). ^1^H-NMR (CDCl_3_. δ/ppm. 300 MHz): 6.99-7.01 (d, 1H, O=C-C=CH-); 5.81-5.83 (d, 1H, -CH=C-CH-(Me)_2_); 4.09-4.13 (t, 2H, -OCH_2_-); 2.90-2.98 (m, 1H, -CH-(Me)_2_); 2.42-2.48 (t, 2H, O=C-C-CH_2_-); 2.17-2.23 (t, 2H, O=C-C-CH_2_-CH_2_-); 1.63-1.82 (m, 2H, -OCH_2_-CH_2_-); 1.04-1.08 (d, 6H, -CH_3_); 0.94-0.99 (t, 3H, -CH_3_). TOF-ESI-MS (m/z): 209 [M + H] ^+^. Anal. calcd for C_13_H_20_O_2_: C, 74.96; H, 9.68. Found: C, 74.81; H, 9.70.

**Isopropyl 4-isopropylcyclohexa-1, 3-dienecarboxylate (5d, C_13_H_20_O_2_)**

Yield: 4.20 g (67.4%); yellow liquid. IR (cm^-1^): 2963, 2873 (-CH_3_, -CH_2_); 1703 (C=O); 1384, 1373 (-CH-); 1255, 1108 (C-O-C). ^1^H-NMR (CDCl_3_. δ/ppm. 300 MHz): 6.97-6.99 (d, 1H, O=C-C=CH-); 5.80-5.82 (d, 1H, -CH=C-CH-(Me)_2_); 5.12-5.51 (m, 1H, -O-CH-(Me)_2_); 2.88-2.98 (m, 1H, -CH-(Me)_2_); 2.41-2.47 (t, 2H, O=C-C-CH_2_-); 2.16-2.22 (t, 2H, O=C-C-CH_2_-CH_2_-); 1.35, 1.37 (d, 6H, -CH_3_); 1.26, 1.28 (d, 6H, -CH_3_). TOF-ESI-MS (m/z): 209 [M + H] ^+^. Anal. calcd for C_13_H_20_O_2_: C, 74.96; H, 9.68. Found: C, 74.80; H, 9.73.

**Butyl 4-isopropylcyclohexa-1, 3-dienecarboxylate (5e, C_14_H_22_O_2_)**

Yield: 4.71 g (70.8%); yellow liquid. IR (cm^-1^): 2959, 2872 (-CH_3_, -CH_2_); 1708 (C=O); 1251, 1082 (C-O-C). ^1^H- NMR (CDCl_3_. δ/ppm. 300 MHz): 6.98-7.00 (d, 1H, O=C-C=CH-); 5.81-5.83 (d, 1H, -CH=C-CH-(Me)_2_); 4.12-4.17 (t, 2H, -OCH_2_-); 2.89-2.98 (m, 1H, -CH-(Me)_2_); 2.44-2.48 (t, 2H, O=C-C-CH_2_-); 2.16-2.22 (t, 2H, O=C-C-CH_2_-CH_2_-); 1.60-1.67 (m, 2H, -OCH_2_-CH_2_-); 1.35-1.45 (m, 2H, O-CH_2_-CH_2_-CH_2_-); 1.05-1.07 (d, 6H, -CH_3_); 0.92-0.97 (t, 3H, -CH_3_). TOF-ESI-MS (m/z): 223 [M + H] ^+^; 246 [M + Na] ^+^. Anal. calcd for C_14_H_22_O_2_: C, 75.63; H, 9.97. Found: C, 75.60; H, 9.98.

**Isobutyl 4-isopropylcyclohexa-1, 3-dienecarboxylate (5f, C_14_H_22_O_2_)**

Yield: 4.76 g (71.5%); yellow liquid. IR (cm^-1^): 2960, 2873 (-CH_3_, -CH_2_); 1709 (C=O); 1080, 1248 (C-O-C). ^1^H-NMR (CDCl_3_. δ/ppm. 300 MHz): 7.00-7.01 (d, 1H, O=C-C=CH-); 5.81-5.83 (d, 1H, -CH=C-CH-(Me)_2_); 3.92-3.94 (d, 2H, -OCH_2_-); 2.90-2.96 (m, 1H, -CH_2_-CH-(Me)_2_); 2.82-2.85 (m, 1H, -CH-(Me)_2_); 2.42-2.49 (t, 2H, O=C-C-CH_2_-); 2.17-2.23 (t, 2H, O=C-C-CH_2_-CH_2_-); 1.02-1.08 (d, 6H, -CH_3_), 0.95-0.97 (d, 6H, -CH_3_). TOF-ESI-MS (m/z): 223 [M + H] ^+^; 246 [M + Na] ^+^. Anal. calcd for C_14_H_22_O_2_: C, 75.63; H, 9.97. Found: C, 75.62; H, 10.00.

**Tert-butyl 4-isopropylcyclohexa-1, 3-dienecarboxylate (5g, C_14_H_22_O_2_)**

Yield: 4.07 g (61.1%); yellow liquid. IR (cm^-1^): 2963, 2872 (-CH_3_, -CH_2_); 1702 (C=O); 1085, 1275 (C-O-C). ^1^H-NMR (CDCl_3_. δ/ppm. 300 MHz): 6.91-6.93 (d, 1H, O=C-C=CH-); 5.79-5.81 (d, 1H, -CH=C-CH-(Me)_2_); 2.85-2.97 (m, 1H, -CH-(Me)_2_); 2.37-2.43 (t, 2H, O=C-C-CH_2_-); 2.15-2.21 (t, 2H, O=C-C-CH_2_-CH_2_-); 1.49 (s, 9H, -CH_3_); 1.01-1.07 (d, 6H, -CH_3_). TOF-ESI-MS (m/z): 223 [M + H] ^+^, 246 [M + Na] ^+^. Anal. calcd for C_14_H_22_O_2_: C, 75.63; H, 9.97. Found: C, 75.59; H, 9.99.

**Sec-butyl 4-isopropylcyclohexa-1, 3-dienecarboxylate (5h, C_14_H_22_O_2_)**

Yield: 3.48 g (52.3%); yellow liquid. IR (cm^-1^): 2963, 2872 (-CH_3_, -CH_2_); 1702 (C=O); 1085, 1275 (C-O-C). ^1^H-NMR (CDCl_3_. δ/ppm. 300 MHz): 6.91-6.93 (d, 1H, O=C-C=CH-); 5.79-5.81 (d, 1H, -CH=C-CH-(Me)_2_); 4.13 (m, 1H, O=C-O-CH-CH_2_-); 2.85-2.97 (m, 1H, -CH-(Me)_2_); 2.39-2.53 (t, 2H, O=C-C-CH_2_-); 2.12-2.19 (t, 2H, O=C-C-CH_2_-CH_2_-); 1.57 (m, 2H, O=C-O-CH-CH_2_-); 1.40 (d, 3H, -CH_3_), 1.01-1.07 (d, 6H, -CH_3_), 0.96 (t, 3H, -CH_3_). TOF-ESI-MS (m/z): 223 [M + H] ^+^; 246 [M + Na] ^+^. Anal. calcd for C_14_H_22_O_2_: C, 75.63; H, 9.97. Found: C, 75.60; H, 10.00.

**Pentyl 4-isopropylcyclohexa-1, 3-dienecarboxylate (5i, C_15_H_24_O_2_)**

Yield: 4.99 g (70.5%); yellow liquid. IR (cm^-1^): 2963, 2872 (-CH_3_, -CH_2_); 1702 (C=O); 1085, 1275 (C-O-C). ^1^H- NMR (CDCl_3_. δ/ppm. 300 MHz): 6.91-6.93 (d, 1H, O=C-C=CH-); 5.79-5.81 (d, 1H, -CH=C-CH-(Me)_2_); 4.15 (t, 2H, -OCH_2_-); 2.85-2.97 (m, 1H, -CH-(Me)_2_); 2.39-2.53 (t, 2H, O=C-C-CH_2_-); 2.15-2.20 (t, 2H, O=C-C-CH_2_-CH_2_-); 1.57 (m, 2H, -CH_2_-); 1.29-1.33 (m, 4H, -CH_2_-); 1.01-1.07 (d, 6H, -CH_3_), 0.96 (t, 3H, -CH_3_). TOF-ESI-MS (m/z): 237 [M + H] ^+^. Anal. calcd for C_15_H_24_O_2_: C, 76.23; H, 10.24. Found: C, 76.10; H, 10.31.

**Cyclohexyl 4-isopropylcyclohexa-1, 3-dienecarboxylate (5j, C_16_H_24_O_2_)**

Yield: 4.88 g (65.6%), yellow liquid. IR (cm^-1^): 2963, 2872 (-CH_3_, -CH_2_); 1702 (C=O); 1085, 1275 (C-O-C). ^1^H-NMR (CDCl_3_. δ/ppm. 300 MHz): 6.91-6.93 (d, 1H, O=C-C=CH-); 5.79-5.81 (d, 1H, -CH=C-CH-(Me)_2_); 3.91 (m, 1H, -OCH-); 2.85-2.97 (m, 1H, -CH-(Me)_2_); 2.39-2.53 (t, 2H, O=C-C-CH_2_-); 2.16-2.22 (t, 2H, O=C-C-CH_2_-CH_2_-); 1.39-1.80 (m, 10H, -CH_2_-); 1.01-1.07 (d, 6H, -CH_3_). TOF-ESI-MS (m/z): 249 [M + H] ^+^. Anal. calcd for C_16_H_24_O_2_: C, 77.38; H, 9.74. Found: C, 77.10; H, 9.87.

**2-Hydroxyethyl 4-isopropylcyclohexa-1, 3-dienecarboxylate (5k, C_12_H_18_O_3_)**

Yield: 3.48 g (55.3%); yellow liquid. IR (cm^-1^): 3010 (-OH); 2963, 2872 (-CH_3_, -CH_2_); 1702 (C=O); 1085, 1275 (C-O-C). ^1^H-NMR (CDCl_3_. δ/ppm. 300 MHz): 6.91-6.93 (d, 1H, O=C-C=CH-); 5.79-5.81 (d, 1H, -CH=C-CH-(Me)_2_); 4.34 (t, 2H, -OCH_2_-); 3.81 (t, 2H, -OCH_2_-CH_2_-OH); 2.85-2.97 (m, 1H, -CH-(Me)_2_); 2.39-2.53 (t, 2H, O=C-C-CH_2_-); 2.15-2.21 (t, 2H, O=C-C-CH_2_-CH_2_-); 1.01-1.07 (d, 6H, -CH_3_). TOF-ESI-MS (m/z): 211 [M + H] ^+^. Anal. calcd for C_12_H_18_O_3_: C, 68.54; H, 8.63. Found: C, 68.40; H, 8.67.

**2-(2-Hydroxyethoxy) ethyl 4-isopropylcyclohexa-1, 3-dienecarboxylate (5l, C_14_H_22_O_4_)**

Yield: 3.22 g (42.3%); yellow liquid. IR (cm^-1^): 3013 (-OH); 2963, 2872 (-CH_3_, -CH_2_); 1702 (C=O); 1085, 1275 (C-O-C). ^1^H-NMR (CDCl_3_. δ/ppm. 300 MHz): 6.91-6.93 (d, 1H, O=C-C=CH-); 5.79-5.81 (d, 1H, -CH=C-CH-(Me)_2_); 4.32 (t, 2H, -COOCH_2_-); 3.70 (t, 2H, -CH_2_-OH); 3.65-3.56 (t, 4H, -CH_2_-O-CH_2_); 2.85-2.97 (m, 1H, -CH-(Me)_2_); 2.39-2.53 (t, 2H, O=C-C-CH_2_-); 2.16-2.23 (t, 2H, O=C-C-CH_2_-CH_2_-); 1.01-1.07 (d, 6H, -CH_3_). TOF-ESI-MS (m/z): 255 [M + H] ^+^. Anal. calcd for C_14_H_22_O_4_: C, 66.12; H, 8.72. Found: C, 66.10; H, 8.77.

**1-(Funan-2-yl) ethanone oximyl 4-isopropylcyclohexa-1, 3-dienecarboxylate (6a, C_16_H_19_NO_3_)**

Yield: 4.91 g (60.3%); yellow solid; m.p. 63.5 °C. IR (cm^-1^): 1729 (C=O); 1606 (C=N). ^1^H-NMR (CDCl_3_, *δ*/ppm, 300 MHz): 7.54, 7.57 (d, 1H, furan-*α*-C-H); 7.13, 7.14 (d, 1H, O=C-C=CH-); 6.93-6.94 (d, 2H, furan-*β*-C-H); 5.89, 5.90 (d, 1H, -CH=C-CH-(Me)_2_); 2.89-2.91 (m, 1H, -CH-(Me)_2_); 2.52-2.58 (t, 2H, O=C-C-CH_2_-); 2.35 (s, 3H, -N=C-CH_3_); 2.22-2.29 (t, 2H, O=C-C-CH_2_-CH_2_-); 1.07-1.10 (d, 6H, -CH_3_). TOF-ESI-MS (m/z): 295 [M + Na] ^+^. Anal. calcd for C_16_H_19_NO_3_: C, 70.33; H, 6.96; N, 5.13. Found: C, 70.21; H, 7.03; N, 5.18.

**1-(Thiophene-2-yl) ethanone oximyl 4-isopropylcyclohexa-1, 3-dienecarboxylate (6b, C_16_H_19_NO_2_S)**

Yield: 4.79 g (55.3%); yellow solid; m.p. 45.5 °C. IR (cm^-1^): 1738 (C=O); 1584 (C=S). ^1^H-NMR (CDCl_3_, *δ*/ppm, 300 MHz): 8.05, 8.03 (d, 1H, thiophene-*α*-C-H); 7.49, 7.50 (d, 2H, thiophene-*β*-C-H); 7.09, 7.11 (d, 1H, O=C-C=CH-); 5.87, 5.89 (d, 1H, -CH=C-CH-(Me)_2_); 2.50-2.59 (m, H, -CH-(Me)_2_); 2.40-2.43 (t, 2H, O=C-C-CH_2_-); 2.31 (s, 3H, -N=C-CH_3_); 2.23-2.24 (t, 2H, -O=C-C-CH_2_-CH_2_-); 1.06-1.10 (d, 6H, -CH_3_). TOF-ESI-MS (m/z): 289 [M + H] ^+^. Anal. calcd for C_16_H_19_NO_2_S: C, 66.44; H, 6.57; N, 9.69. Found: C, 66.88; H, 6.34; N, 9.48.

**1-(4-Chlorophenyl) ethanone oximyl 4-isopropylcyclohexa-1, 3-dienecarboxylate (6c, C_18_H_20_ClNO_2_)**

Yield: 6.26 g (65.3%); yellow solid; m.p. 45.5 °C. IR (cm^-1^): 1734 (C=O); 1592 (C=N). ^1^H-NMR (CDCl_3_, *δ*/ppm, 300 MHz): 7.70, 7.72 (d, 2H, *o*-C-H); 7.33-7.36 (d, 2H, *m*-C-H); 7.13, 7.14 (d, 1H, O=C-C=CH-); 5.91, 5.92 (d, 1H, -CH=C-CH-(Me)_2_); 2.53-2.55 (m, 1H, -CH-(Me)_2_); 2.40-2.41 (t, 2H, O=C-C-CH_2_-); 2.30 (s, 3H, -N=C-CH_3_); 2.20-2.23 (t, 2H, -O=C-C-CH_2_-CH_2_-); 1.04-1.08 (d, 6H, CH_3_). TOF-ESI-MS (m/z): 317 [M + H] ^+^. Anal. calcd for C_18_H_20_ClNO_2_: C, 68.03; H, 6.30; N: 4.41. Found: C, 67.86; H, 6.31; N, 4.51.

**1-(*p*-Tolyl) ethanone oximyl 4-isopropylcyclohexa-1, 3-dienecarboxylate** (**6d,** **C_19_H_23_NO_2_)**

Yield: 5.82 g (65.3%); yellow solid; m.p. 33.0 °C. IR (cm^-1^): 1734 (C=O); 1592 (C=N). ^1^H-NMR (CDCl_3_, *δ*/ppm, 300 MHz): 7.64, 7.79 (d, 2H, *o*-C-H), 7.51, 7.54 (d, 2H, *m*-C-H); 7.12, 7.17 (d, 1H, O=C-C=CH-), 5.87, 5.88 (d, 1H, -CH=C-CH-(Me)_2_); 2.50-2.58 (m, 1H, -CH-(Me)_2_); 2.40-2.41 (t, 2H, O=C-C-CH_2_-); 2.21-2.23 (t, 2H, -O=C-C-CH_2_-CH_2_-); 2.27 (s, 3H, Ar-H); 1.80 (s, 3H, -N=C-CH_3_); 1.04-1.08 (d, 6H, -CH_3_). TOF-ESI-MS (m/z): 297 [M + H] ^+^. Anal. calcd for C_19_H_23_NO_2_: C, 76.77; H, 7.74; N, 4.71. Found: C, 75.86; H, 8.15; N, 5.21.

**Dicyclohexylmethanone oximyl 4-isopropylcyclohexa-1, 3-dienecarboxylate** **(6e, C_23_H_35_NO_2_)**

Yield: 5.92 g (55.3%), yellow solid; m.p. 30.3 °C. IR (cm^-1^): 1735 (C=O); 1608 (C=N). ^1^H-NMR (CDCl_3_, *δ*/ppm, 300 MHz): 7.04, 7.05 (d, 1H, O=C-C=CH-); 5.83, 5.85 (d, 1H, -CH=C-CH-(Me)_2_); 2.51-2.57 (m, 1H, -CH-(Me)_2_); 2.35-2.40 (t, 2H, O=C-C-CH_2_-); 2.20-2.26 (t, 2H, -O=C-C-CH_2_-CH_2_-); 2.06-2.18 (m, 2H, cyclehexyl-CH); 1.66-1.78 (m, 20H, cyclehexyl-CH_2_); 1.02-1.06 (d, 6H, -CH_3_). TOF-ESI-MS (m/z): 261 [M + H] ^+^. Anal. calcd for C_23_H_35_NO_2_: C, 77.31; H, 9.80; N, 3.92. Found: C, 77.65; H, 9.63; N, 3.35.

**Picolinaldehyde oximyl 4-isopropylcyclohexa-1, 3-dienecarboxylate (6f, C_16_H_18_N_2_O_2_)**

Yield: 4.48 g (55.3%); white solid; m.p. 68.0 °C. IR (cm^-1^): 1728 (C=O); 1646 (C=N). ^1^H- NMR (CDCl_3_, *δ*/ppm, 300 MHz): 8.66-8.68 (d, 1H, pyridine-*α*-C-H); 7.75-7.80 (m, 1H, pyridine-*γ*-C-H); 7.35-7.37 (m, 2H, pyridine-*β*-C-H); 7.18, 7.20 (d, 1H, O=C-C=CH-), 5.83, 5.85 (d, 1H, -CH=C-CH-(Me)_2_); 2.89 (s, 1H, -N=C-H); 2.40-2.45 (m, 1H, -CH-(Me)_2_); 2.40-2.41 (t, 2H, O=C-C-CH_2_-); 2.20-2.23 (t, 2H, -O=C-C-CH_2_-CH_2_-); 1.04-1.08 (d, 6H, -CH_3_). TOF-ESI-MS (m/z): 270 [M + H] ^+^. Anal. calcd for C_16_H_18_N_2_O_2_: C, 71.11; H, 6.67; N, 10.37. Found: C, 71.22; H, 6.87; N, 10.58.

**Propan-2-one oximyl 4-isopropylcyclohexa-1, 3-dienecarboxylate (6g, C_13_H_19_NO_2_)**

Yield: 3.67 g (55.3%); white solid; m.p. 39.1 °C. IR (cm^-1^): 1735 (C=O); 1643 (C=N). ^1^H- NMR (CDCl_3_, *δ*/ppm, 300 MHz): 7.04, 7.06 (d, 1H, O=C-C=CH-); 5.83, 5.85 (d, 1H, -CH=C-CH-(Me)_2_); 2.42-2.54 (m, 1H, -CH-(Me)_2_); 2.39-2.41 (t, 2H, O=C-C-CH_2_-); 2.20-2.26 (t, 2H, -O=C-C-CH_2_-CH_2_-); 2.00 (s, 6H, -N=C-(Me-H)_2_); 1.02-1.06 (d, 6H, -CH_3_). TOF-ESI-MS (m/z): 221 [M + H] ^+^. Anal. calcd for C_13_H_19_NO_2_: C, 70.59; H, 8.60; N, 6.33. Found: C, 71.11; H, 8.68; N, 5.73.

**Butan-2-one oximyl 4-isopropylcyclohexa-1, 3-dienecarboxylate (6h, C_14_H_21_NO_2_)**

Yield: 4.50 g (60.3%); yellow solid; m.p. 37.0 °C. IR (cm^-1^): 1729 (C=O); 1643 (C=N). ^1^H- NMR (CDCl_3_, *δ*/ppm, 300 MHz): 7.04, 7.07 (d, 1H, O=C-C=CH-); 5.84, 5.86 (d, 1H, -CH=C-CH-(Me)_2_); 2.57-2.60 (m, 1H, -CH-(Me)_2_); 2.39-2.41 (t, 2H, O=C-C-CH_2_-); 2.15-2.17 (t, 2H, -O=C-C-CH_2_-CH_2_-); 2.00 (s, 3H, -N=C-CH_3_); 1.37-1.41 (m, 2H, -N=C-CH_2_-); 1.03-1.05 (d, 6H, -CH_3_); 0.90-1.00 (t, 3H, -N=C-CH_2_-CH_3_). TOF-ESI-MS (m/z): 236 [M + H] ^+^; 258 [M + Na] ^+^. Anal. calcd for C_14_H_21_NO_2_: C, 71.49; H, 8.94; N, 5.96. Found: C, 70.79; H, 9.00; N, 6.60.

**(*E*)-Benzaldehyde oximyl 4-isopropylcyclohexa-1, 3-dienecarboxylate (6i, C_17_H_19_NO_2_)**

Yield: 3.25 g (40.3%); yellow solid; m.p. 67.8 °C. IR (cm^-1^): 3395 (H-C=N); 1725 (C=O); 1646 (C=N). ^1^H-NMR (CDCl_3_, *δ*/ppm, 300 MHz): 8.43 (s, 1H, -N=CH); 7.76, 7.78 (d, 2H, *o*-C-H); 7.33, 7.43 (m, 3H, *m*, *p*-C-H); 7.15, 7.16 (d, 1H, O=C-C=CH-); 5.84, 5.85 (d, 1H, -CH=C-CH-(Me)_2_); 2.52-2.58 (m, 1H, -CH-(Me)_2_); 2.40-2.41 (t, 2H, O=C-C-CH_2_-); 2.21-2.29 (t, 2H, -O=C-C-CH_2_-CH_2_-); 1.04-1.10 (d, 6H, -CH_3_). TOF-ESI-MS (m/z): 269 [M + H] ^+^. Anal. calcd for C_17_H_19_NO_2_: C, 76.12; H, 7.06 ; N, 5.20. Found: C, 75.92; H, 7.16; N, 5.30.

**Acetophenone oximyl 4-isopropylcyclohexa-1, 3-dienecarboxylate (6j, C_18_H_21_NO_2_)**

Yield: 5.06 g (59.6%); yellow solid; m.p. 61.8 °C. IR (cm^-1^): 1727 (C=O); 1641 (C=N). ^1^H- NMR (CDCl_3_, *δ*/ppm, 300 MHz): 7.77, 7.79 (d, 2H, *o*-C-H); 7.37-7.42 (m, 3H, *m, p*-C-H); 7.14, 7.17 (d, 1H, O=C-C=CH-); 5.83, 5.85 (d, 1H, -CH=C-CH-(Me)_2_); 2.52-2.57 (m, 1H, -CH-(Me)_2_); 2.40-2.42 (t, 2H, O=C-C-CH_2_-); 2.21-2.23 (t, 2H, -O=C-C-CH_2_-CH_2_-); 2.30 (s, 3H, -N=C-CH_3_); 1.04-1.10 (d, 6H, -CH_3_). TOF-ESI-MS (m/z): 283 [M + H] ^+^. Anal. calcd for C_18_H_21_NO_2_: C, 76.33; H, 7.42; N, 4.95. Found: C, 76.35; H, 7.57; N, 4.78.

**1-(Thiophene-2-yl) methanone oximyl 4-isopropylcyclohexa-1, 3-dienecarboxylate (6k, C_15_H_17_NO_2_S)**

Yield: 4.22 g (51.2%); yellow solid; m.p. 46.7 °C. IR (cm^-1^): 1738 (C=O); 1584 (C=S). ^1^H-NMR (CDCl_3_, *δ*/ppm, 300 MHz): 8.11 (s, 1H, -N=C-H); 8.05, 8.03 (d, 1H, thiophene-*α*-C-H); 7.49, 7.50 (d, 2H, thiophene-*β*-C-H); 7.09, 7.11 (d, 1H, O=C-C=CH-); 5.87, 5.89 (d, 1H, -CH=C-CH-(Me)_2_); 2.50-2.59 (m, 1H, -CH-(Me)_2_); 2.40-2.43 (t, 2H, O=C-C-CH_2_-); 2.23-2.24 (t, 2H, -O=C-C-CH_2_-CH_2_-); 1.06-1.10 (d, 6H, -CH_3_). TOF-ESI-MS (m/z): 276 [M + H] ^+^. Anal. calcd for C_15_H_17_NO_2_S: C, 65.45; H, 6.18; N, 5.09. Found: C, 65.88; H, 6.04; N, 5.48.

**1-(Funan-2-yl) methanone oximyl 4-isopropylcyclohexa-1, 3-dienecarboxylate (6l, C_15_H_17_NO_3_)**

Yield: 4.26 g (54.8%); yellow solid; m.p. 60.5 °C. IR (cm^-1^): 1729 (C=O); 1606 (C=N). ^1^H-NMR (CDCl_3_, *δ*/ppm, 300 MHz): 8.05 (s, 1H, -N=C-H); 7.54, 7.57 (d, 1H, furan-*α*-C-H); 7.14, 7.13 (d, 1H, O=C-C=CH-); 6.93-6.94 (d, 2H, furan-*β*-C-H); 5.89, 5.90 (d, 1H, -CH=C-CH-(Me)_2_); 2.89-2.91 (m, 1H, -CH-(Me)_2_); 2.52-2.58 (t, 2H, O=C-C-CH_2_-); 2.22-2.29 (t, 2H, -O=C-C-CH_2_-CH_2_-); 1.07-1.10 (d, 6H, -CH_3_). TOF-ESI-MS (m/z): 283 [M + Na] ^+^. Anal. calcd for C_15_H_17_NO_3_: C, 69.50; H, 6.56; N, 5.41. Found: C, 70.01; H, 6.43; N, 5.28.

**1-(Benzo[*d*] oxazole -2-yl) methanone oximyl 4-isopropylcyclohexa-1, 3-dienecarboxylate (6m, C_18_H_18_N_2_O_3_)**

Yield: 5.55 g (59.7%); yellow solid; m.p. 79.1 °C. IR (cm^-1^): 1729 (C=O); 1606 (C=N). ^1^H-NMR (CDCl_3_, *δ*/ppm, 300 MHz): 8.05 (s, 1H, -N=C-H); 7.54, 7.57 (m, 4H, Ar-H); 7.14, 7.13 (d, 1H, O=C-C=CH-); 5.89, 5.90 (d, 1H, -CH=C-CH-(Me)_2_); 2.89-2.91 (m, 1H, -CH-(Me)_2_); 2.52-2.58 (t, 2H, O=C-C-CH_2_-); 2.22-2.29 (t, 2H, -O=C-C-CH_2_-CH_2_-); 1.07-1.10 (d, 6H, -CH_3_). TOF-ESI-MS (m/z): 311 [M + H] ^+^. Anal. calcd for C_18_H_18_N_2_O_3_: C, 69.68; H, 5.81; N, 9.03. Found: C, 70.01; H, 5.93; N, 9.28.

**1-(Pyrrole-2-yl) ethanone oximyl 4-isopropylcyclohexa-1, 3-dienecarboxylate (6n, C_16_H_20_N_2_O_2_)**

Yield: 5.30 g (65.0%); yellow solid; m.p. 45.1 °C. IR (cm^-1^): 1729 (C=O); 1606 (C=N). ^1^H-NMR (CDCl_3_, *δ*/ppm, 300 MHz): 7.00-7.10 (t, 1H, pyrrole-*α*-C-H); 7.14, 7.13 (d, 1H, O=C-C=CH-); 6.03-6.14 (m, 2H, pyrrole-*β*-C-H); 5.89, 5.90 (d, 1H, -CH=C-CH-(Me)_2_); 2.89-2.91 (m, 1H, -CH-(Me)_2_); 2.52-2.58 (t, 2H, O=C-C-CH_2_-); 2.00 (s, 3H, -N=C-CH_3_); 2.22-2.29 (t, 2H, -O=C-C-CH_2_-CH_2_-); 1.07-1.10 (d, 6H, -CH_3_). TOF-ESI-MS (m/z): 273 [M + H] ^+^. Anal. calcd for C_16_H_20_N_2_O_2_: C, 70.59; H, 7.35; N, 10.29. Found: C, 70.31; H, 7.23; N, 10.18.


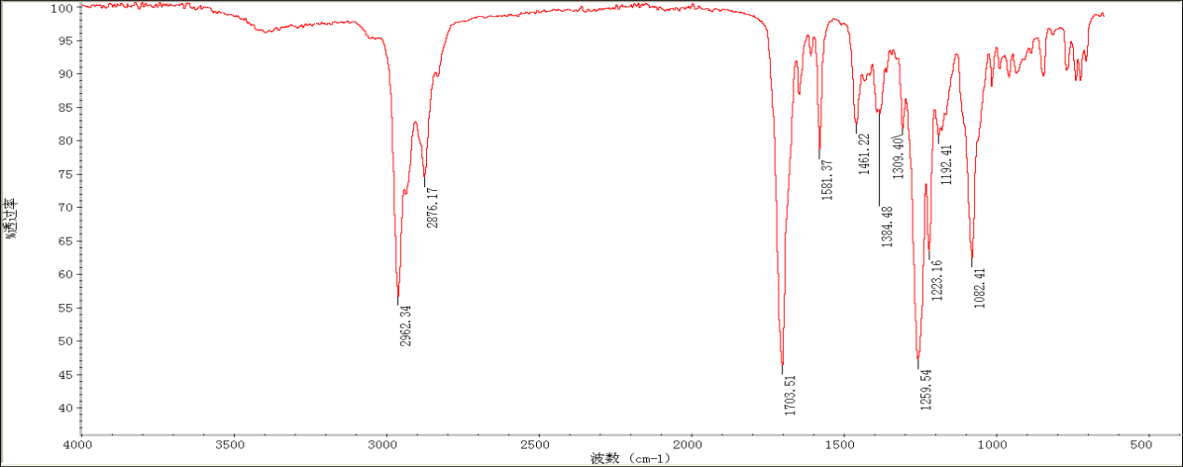


**Figure S1.** FT-IR spectrum of the compound **5a**


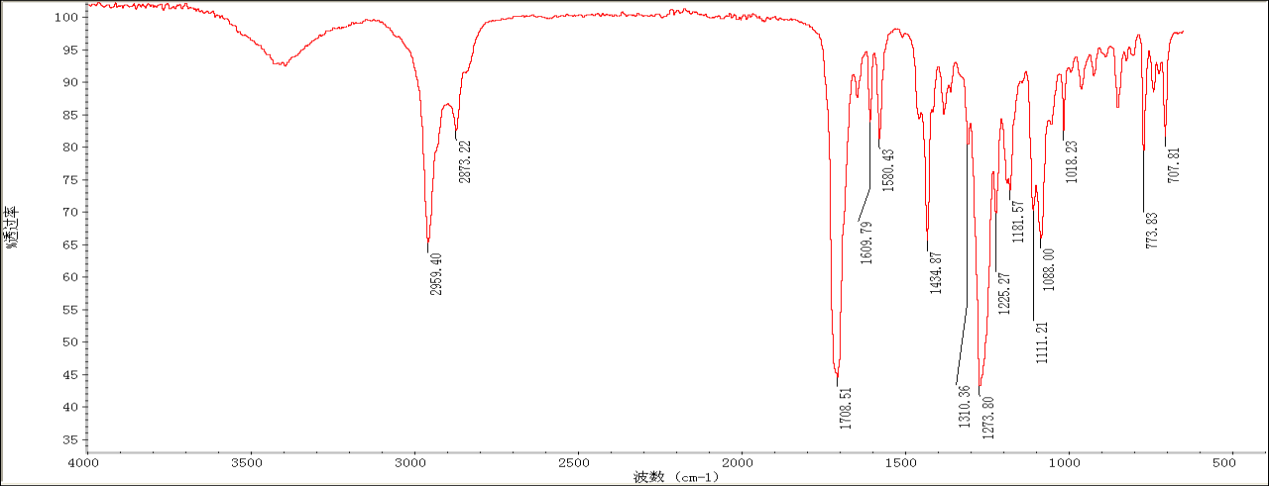


**Figure S2.** FT-IR spectrum of the compound **5b**


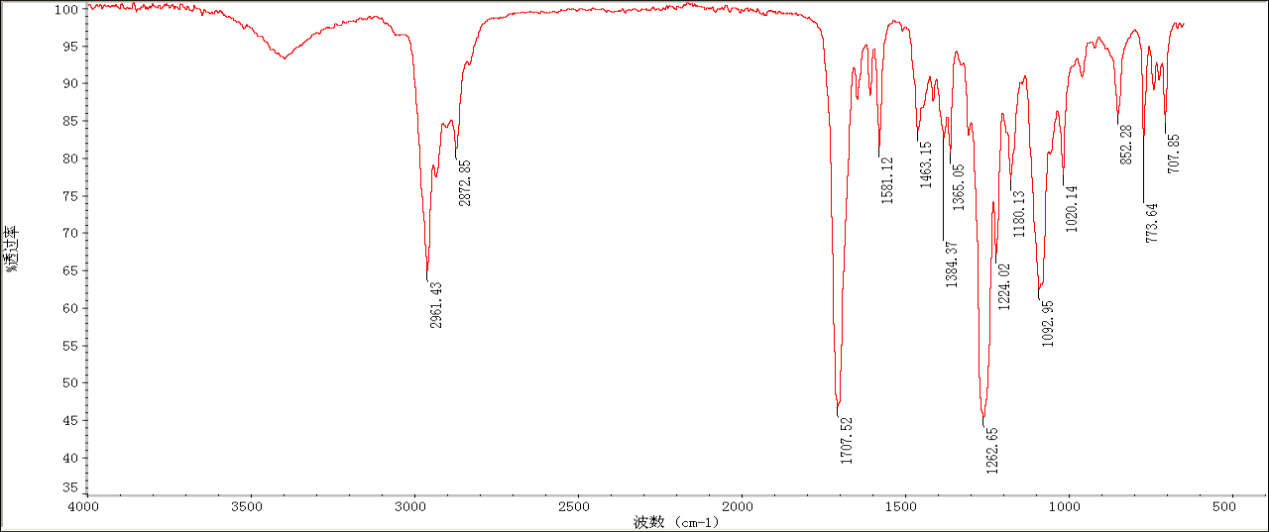


**Figure S3.** FT-IR spectrum of the compound **5c**


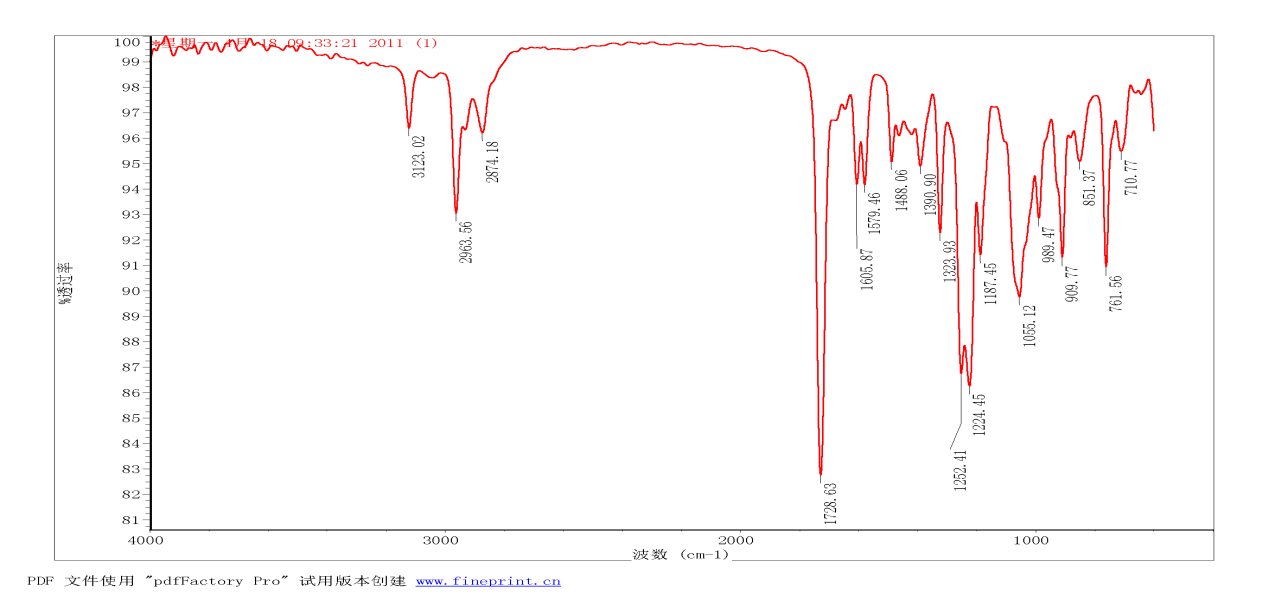


**Figure S4.** FT-IR spectrum of the compound **6a**


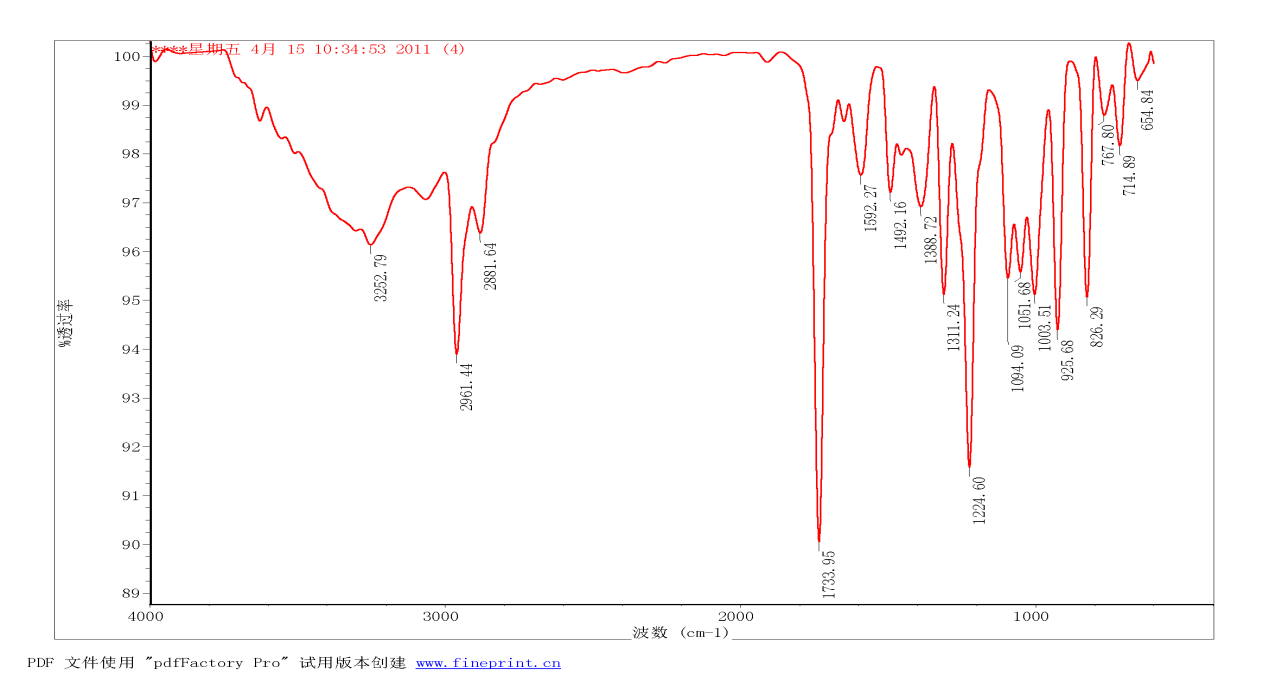


**Figure S5.** FT-IR spectrum of the compound **6c**


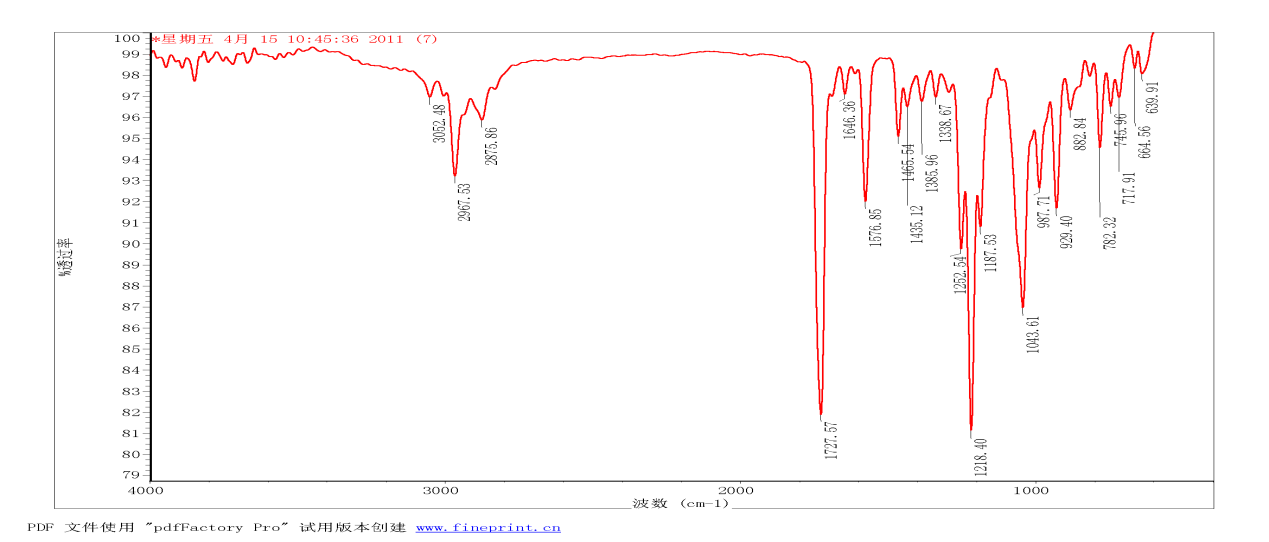


**Figure S6.** FT-IR spectrum of the compound **6f**


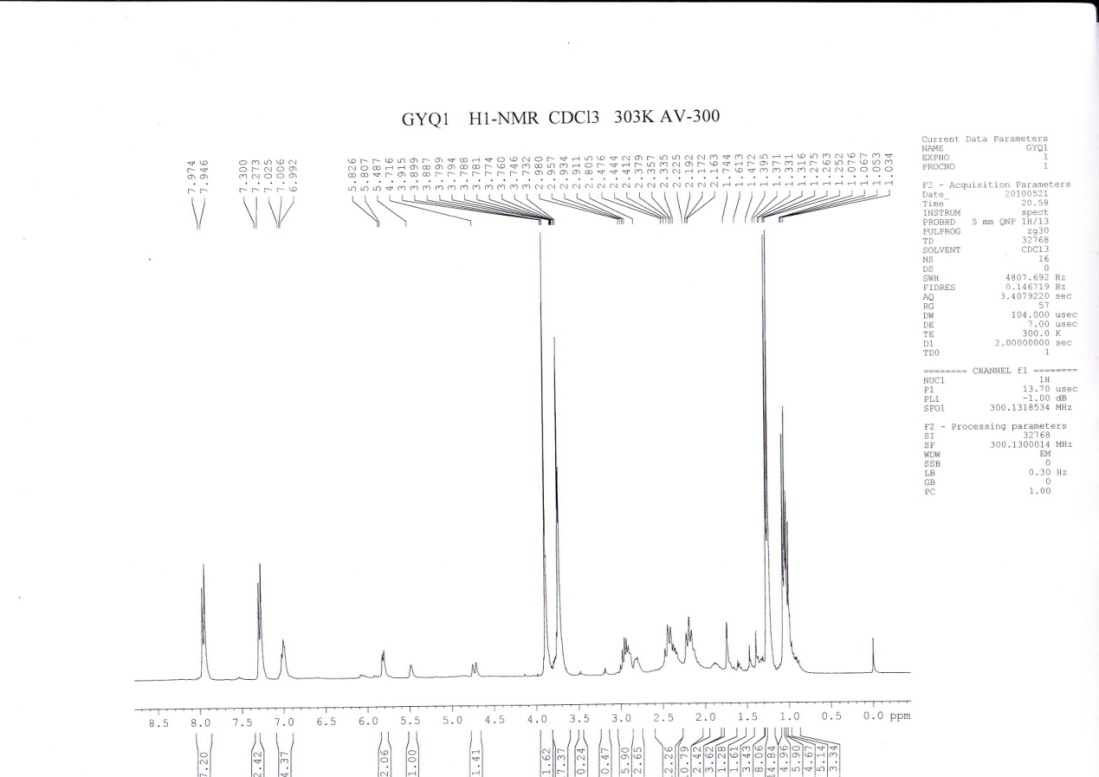


**Figure S7.** ^1^H NMR spectrum of the compound **5a**


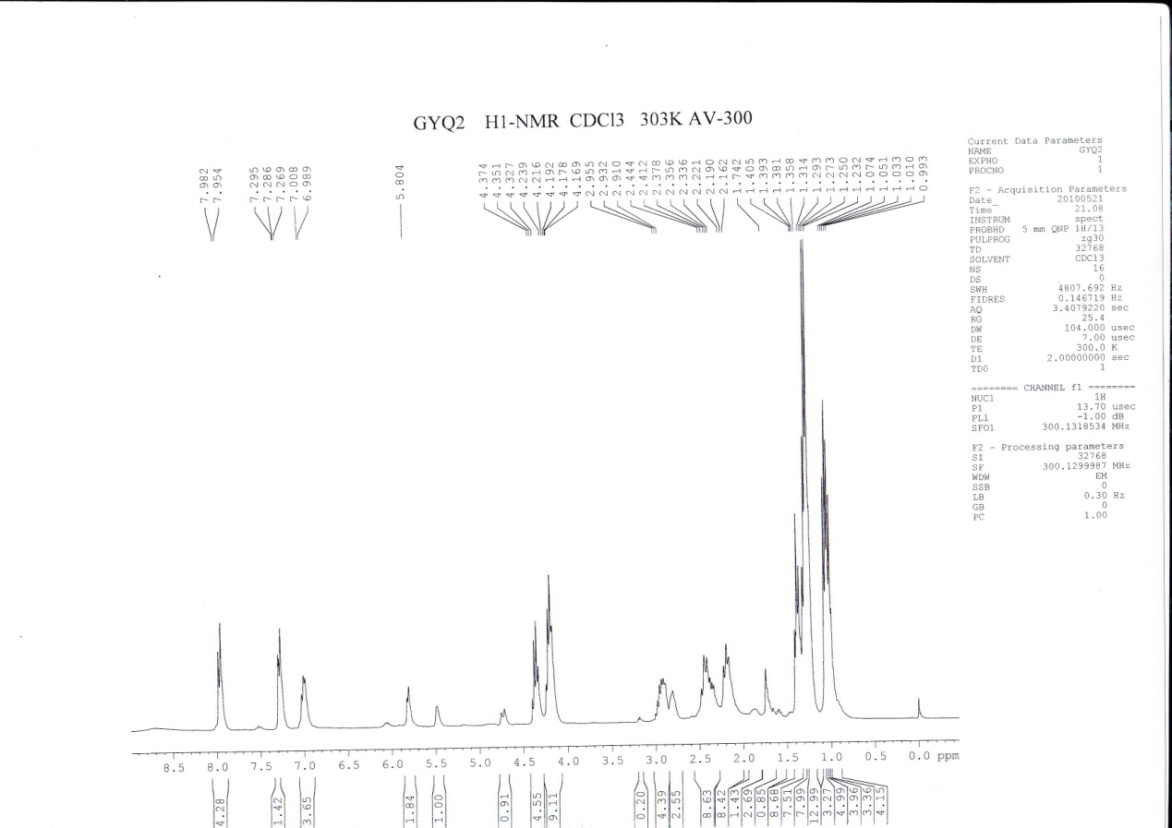


**Figure S8.** ^1^H NMR spectrum of the compound **5b**


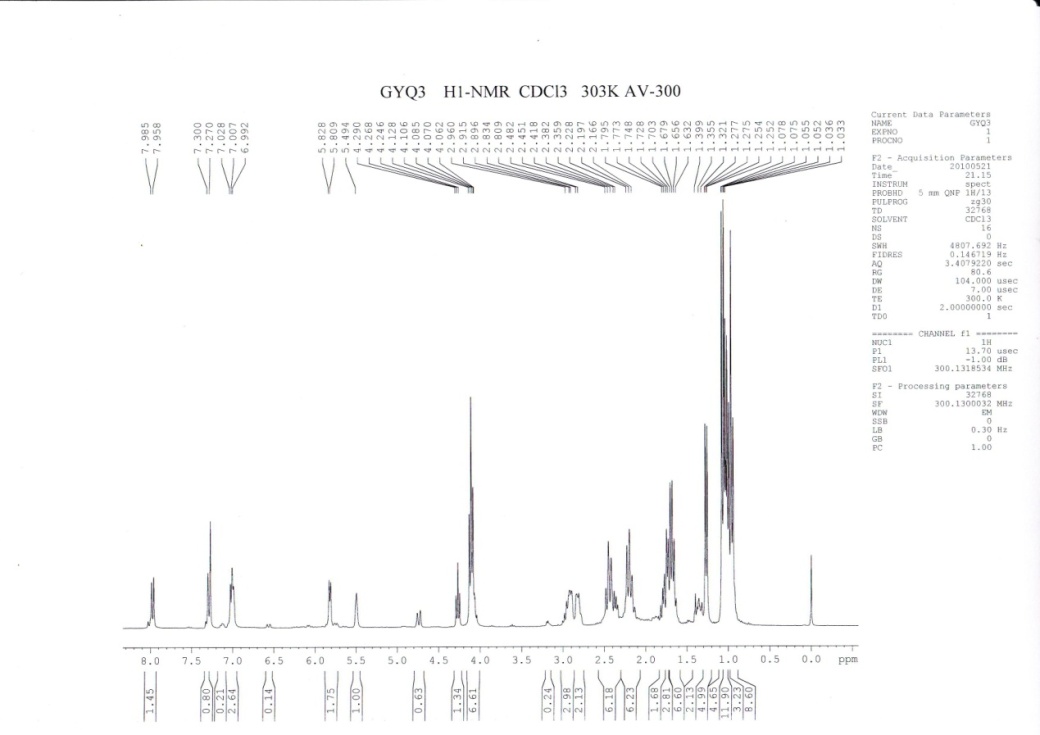


**Figure S9.** ^1^H NMR spectrum of the compound **5c**

**
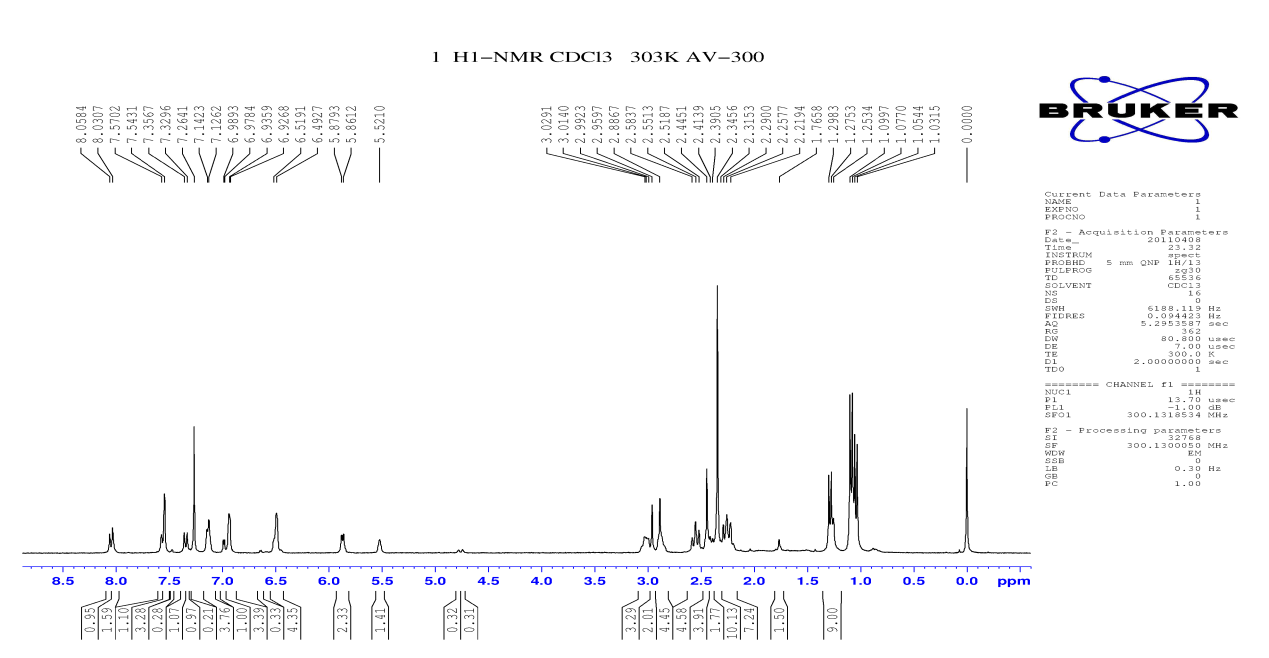
**

**Figure S10.** ^1^H NMR spectrum of the compound **6a**

**
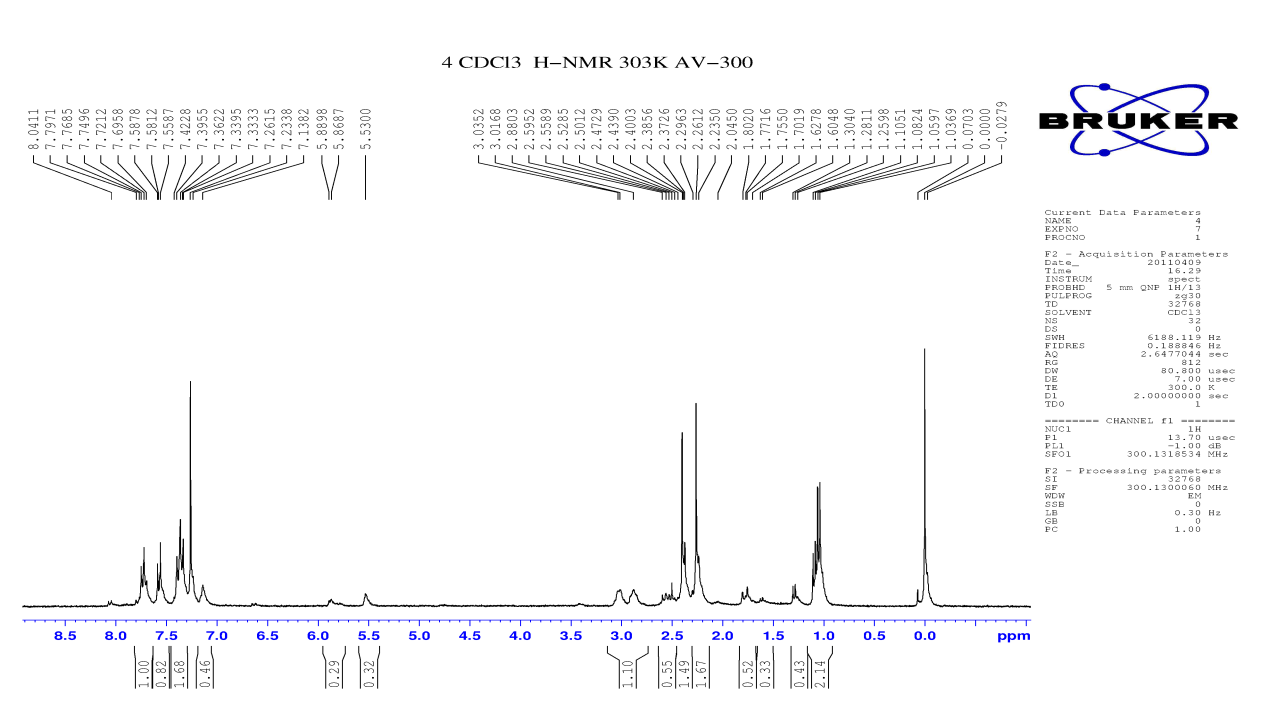
**

**Figure S11.** ^1^H NMR spectrum of the compound **6c**

**
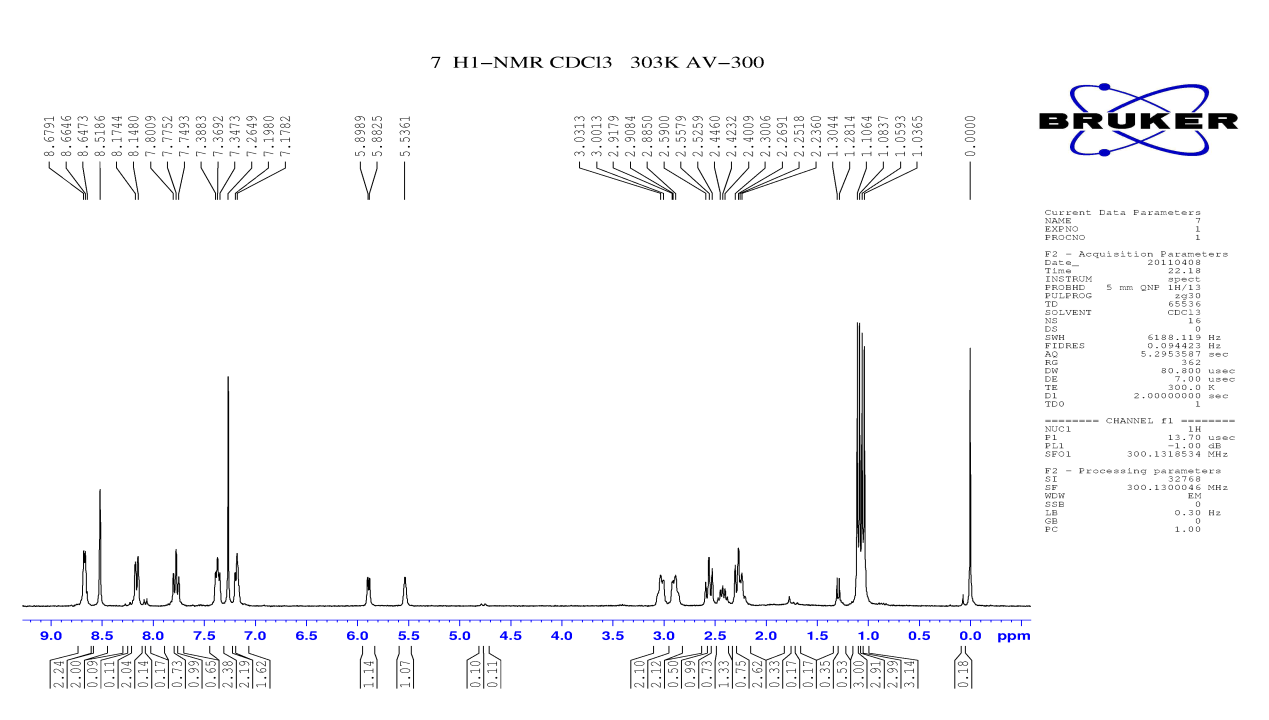
**

**Figure S12.** ^1^H NMR spectrum of the compound **6f**


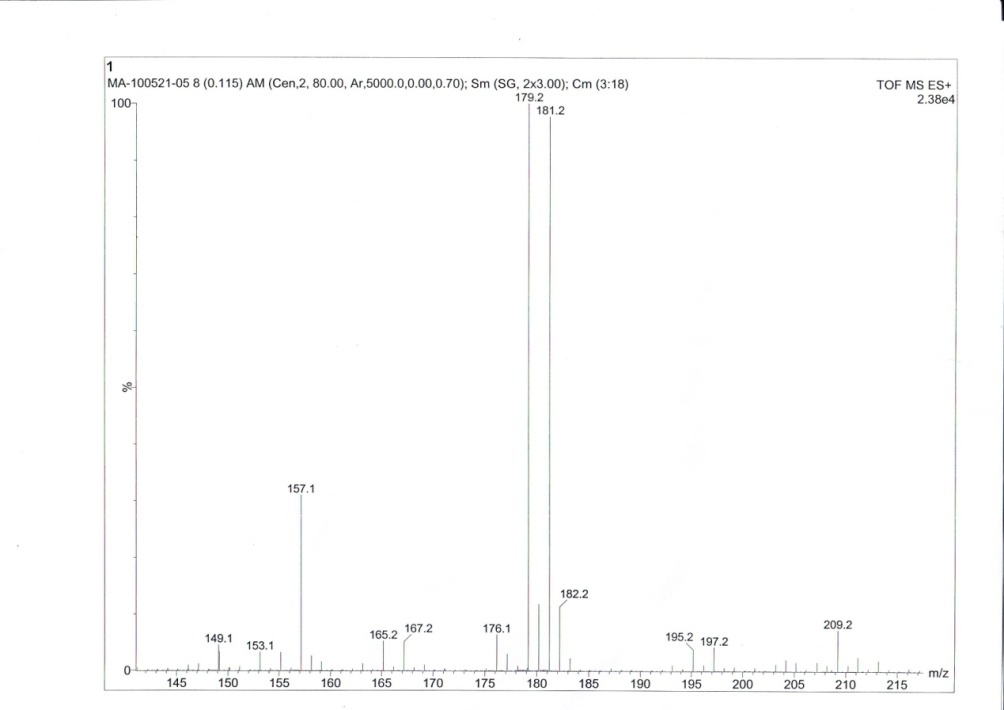


**Figure S13.** ESI-MS spectrum of the compound **5a**


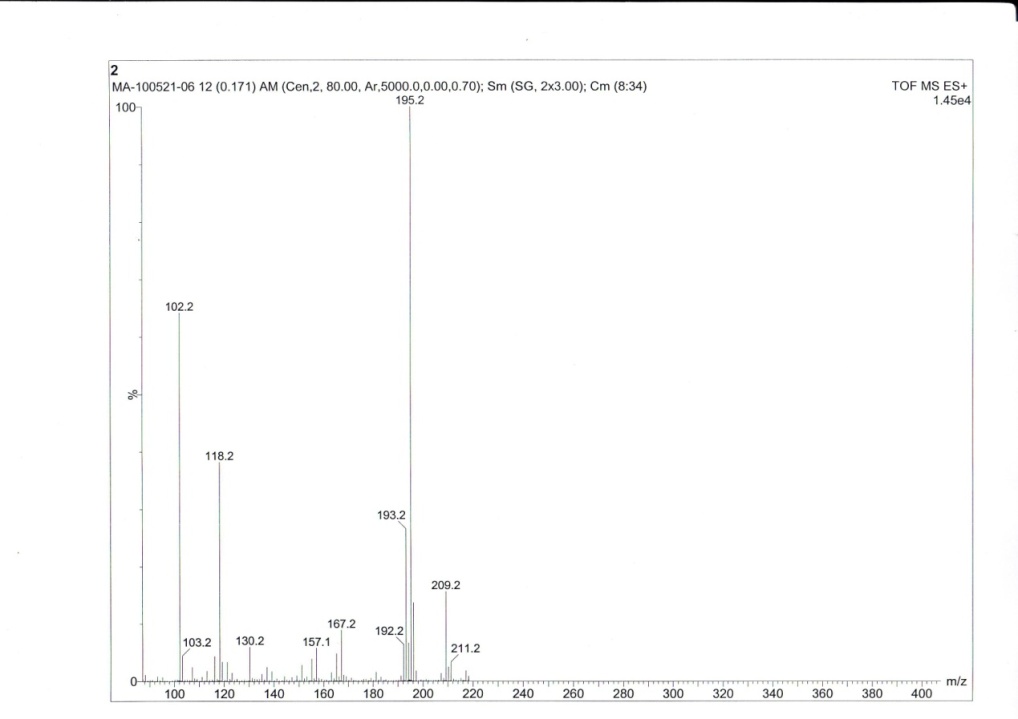


**Figure S14.** ESI-MS spectrum of the compound **5b**


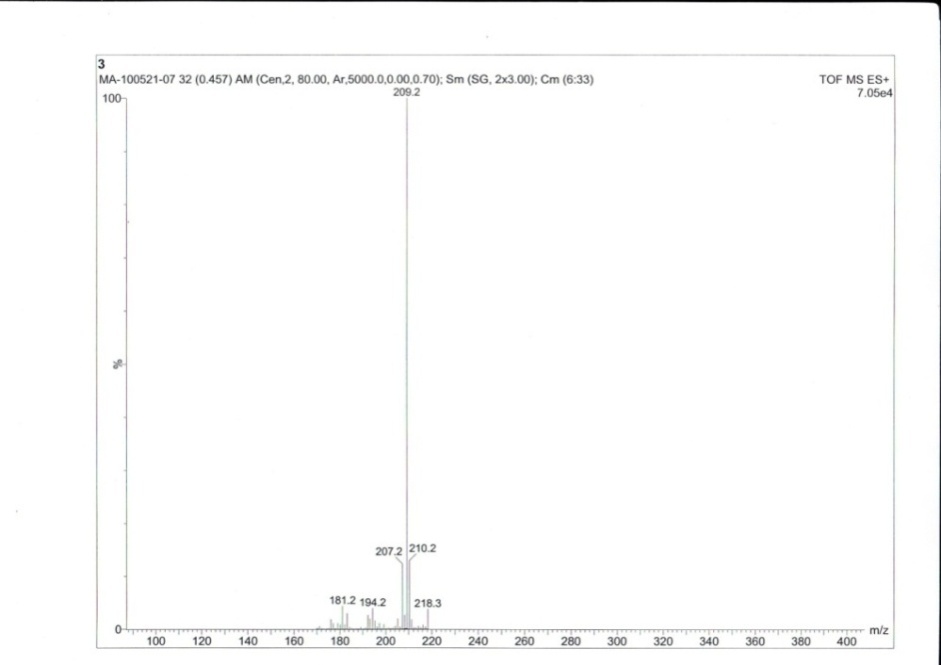


**Figure S15.** ESI-MS spectrum of the compound **5c**
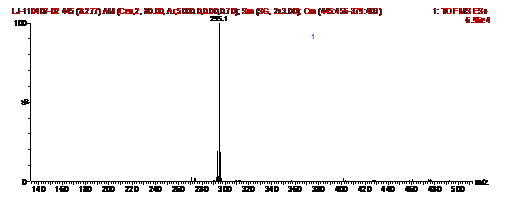


**Figure S16.** ESI-MS spectrum of the compound **6a**

**Figure S17.** ESI-MS spectrum of the compound **6c**

**Figure S18.** ESI-MS spectrum of the compound **6f**

**2. Elucidation of the Herbicidal Activity of the Synthesized Compounds**

**Table S1 Herbicidal activity of compounds 5a-l and 6a-n (200 µg/mL, inhibition percentage/%)**

| Compd. | EC | AR | BC |  |  | Compd. | EC | AR | BC |
| --- | --- | --- | --- | --- | --- | --- | --- | --- | --- |
| **3** | 80 | 30 | 9 |  |  | **6a** | 100 | 34 | 13 |
| **5a** | 100 | 30 | 10 |  |  | **6b** | 100 | 26 | 15 |
| **5b** | 100 | 30 | 10 |  |  | **6c** | 95 | 18 | 0 |
| **5c** | 100 | 30 | 10 |  |  | **6d** | 93 | 18 | 0 |
| **5d** | 100 | 30 | 10 |  |  | **6e** | 85 | 0 | 0 |
| **5e** | 96 | 23 | 5.5 |  |  | **6f** | 100 | 20 | 0 |
| **5f** | 90 | 22 | 0 |  |  | **6g** | 90 | 0 | 0 |
| **5g** | 100 | 24 | 0 |  |  | **6h** | 88 | 0 | 0 |
| **5h** | 91 | 22 | 0 |  |  | **6i** | 98 | 19 | 0 |
| **5i** | 92 | 24 | 0 |  |  | **6j** | 93 | 17 | 0 |
| **5j** | 88 | 0 | 0 |  |  | **6k** | 100 | 25 | 14 |
| **5k** | 75 | 0 | 0 |  |  | **6l** | 100 | 26 | 14 |
| **5l** | 80 | 0 | 0 |  |  | **6m** | 100 | 24 | 15 |
| **Sulfentrazone** | 100 | 100 | 100 |  |  | **6n** | 100 | 20 | 15 |

EC, *Echinochloa crus-galli*; AR, *Amaranthus retroﬂexus L*; BC, *Brassica campestris L*.

**Table S2 Herbicidal activity and structure descriptors of the title compounds**

| No. | Compd. | log IC_50_ | Structure descriptor | | | |
| --- | --- | --- | --- | --- | --- | --- |
|  |  |  | **ΔH_f_** ^a^ | **P**_µµ_ ^b^ | **µ**^c^ | $\mathbf{q}_{\mathbf{max}}^{\mathbf{O}}$^d^ |
| 1 | **5a** | 1.300 | -2.9161 | 1.9142 | 2.4140 | -0.2785 |
| 2 | **5b** | 1.323 | -2.8192 | 1.9141 | 2.2530 | -0.2801 |
| 3 | **5c** | 1.343 | -2.7703 | 1.9141 | 2.2300 | -0.2792 |
| 4 | **5d** | 1.364 | -2.6502 | 1.9136 | 2.0570 | -0.2822 |
| 5 | **5e** | 1.407 | -2.7140 | 1.9141 | 2.2990 | -0.2786 |
| 6 | **5f** | 1.410 | -2.6633 | 1.9141 | 2.2660 | -0.2777 |
| 7 | **5g** | 1.379 | -2.4761 | 1.9136 | 2.1090 | -0.2829 |
| 8 | **5h** | 1.404 | -2.5979 | 1.9136 | 2.0880 | -0.2803 |
| 9 | **5i** | 1.425 | -2.6805 | 1.9141 | 2.3080 | -0.2784 |
| 10 | **5j** | 1.408 | -2.5680 | 1.9141 | 2.2170 | -0.2797 |
| 11 | **5k** | 1.812 | -4.1400 | 1.9751 | 3.9580 | -0.2854 |
| 12 | **5l** | 1.800 | -4.5939 | 1.9757 | 2.3360 | -0.2832 |
| 13 | **6a** | 1.277 | -0.2646 | 1.9118 | 4.4200 | -0.0989 |
| 14 | **6b** | 1.243 | 0.3479 | 1.9118 | 3.9550 | -0.2330 |
| 15 | **6c** | 1.337 | 0.0355 | 1.9819 | 2.8650 | -0.2392 |
| 16 | **6d** | 1.325 | 0.0168 | 1.9119 | 4.3940 | -0.2364 |
| 17 | **6e** | 1.431 | -1.2578 | 1.9119 | 3.8440 | -0.2422 |
| 18 | **6f** | 1.295 | 0.6836 | 1.9118 | 5.8110 | -0.2477 |
| 19 | **6g** | 1.375 | -0.7960 | 1.9119 | 4.0980 | -0.2431 |
| 20 | **6h** | 1.369 | -0.9014 | 1.9120 | 3.9460 | -0.2420 |
| 21 | **6i** | 1.306 | 0.3176 | 1.9118 | 4.3320 | -0.2437 |
| 22 | **6j** | 1.305 | 0.2108 | 1.9119 | 4.0700 | -0.2370 |
| 23 | **6k** | 1.235 | 0.4782 | 1.9119 | 4.2410 | -0.2397 |
| 24 | **6l** | 1.204 | -0.1730 | 1.9118 | 4.8190 | -0.0981 |
| 25 | **6m** | 1.196 | 0.1829 | 1.9127 | 3.3730 | -0.1051 |
| 26 | **6n** | 1.281 | -0.2646 | 1.9118 | 4.4200 | -0.0989 |
|  |  |  |  |  |  |  |
|  |  |  |  |  |  |  |
|  |  |  |  |  |  |  |
|  |  |  |  |  |  |  |
|  |  |  |  |  |  |  |

**3. Validation of the QSAR Model**

**Table S3 Internal validation of the QSAR model**

| Training set | *N* | *R*^2^ | *F* | *S*^2^ | Test set | *N* | *R*^2^ | *F* | *S*^2^ |
| --- | --- | --- | --- | --- | --- | --- | --- | --- | --- |
| A+B | 18 | 0.9458 | 86.88 | 0.0014 | C | 8 | 0.9415 | 86.76 | 0.0013 |
| B+C | 17 | 0.9411 | 86.71 | 0.0015 | A | 9 | 0.9431 | 86.35 | 0.0015 |
| A+C | 17 | 0.9417 | 86.57 | 0.0015 | B | 9 | 0.9470 | 86.75 | 0.0013 |
| Average |  | 0.9429 | 86.72 | 0.0015 | Average |  | 0.9439 | 86.62 | 0.0014 |
